# *N*^1^-methylpseudouridylation of mRNA causes +1 ribosomal frameshifting

**DOI:** 10.1038/s41586-023-06800-3

**Published:** 2023-12-06

**Authors:** Thomas E. Mulroney, Tuija Pöyry, Juan Carlos Yam-Puc, Maria Rust, Robert F. Harvey, Lajos Kalmar, Emily Horner, Lucy Booth, Alexander P. Ferreira, Mark Stoneley, Ritwick Sawarkar, Alexander J. Mentzer, Kathryn S. Lilley, C. Mark Smales, Tobias von der Haar, Lance Turtle, Susanna Dunachie, Paul Klenerman, James E. D. Thaventhiran, Anne E. Willis

**Affiliations:** 1 MRC Toxicology Unit, University of Cambridge, Cambridge, UK; 2 Wellcome Centre for Human Genetics, University of Oxford, Oxford, UK; 3 Department of Biochemistry, University of Cambridge, Cambridge, UK; 4 School of Biosciences, Division of Natural Sciences, University of Kent, Canterbury, UK; 5 National Institute for Bioprocessing Research and Training, University College Dublin, Foster Avenue, Mount Merrion, Dublin, Ireland; 6 NIHR Health Protection Research Unit for Emerging and Zoonotic Infections, Institute of Infection, Veterinary and Ecological Sciences, University of Liverpool, Liverpool, UK; 7 NIHR Oxford Biomedical Research Centre, Oxford University Hospitals NHS Foundation Trust, Oxford, UK; 8 NDM Centre for Global Health Research, Nuffield Department of Medicine, University of Oxford, Oxford, UK; 9 Mahidol-Oxford Tropical Medicine Research Unit, Mahidol University, Bangkok, Thailand; 10 Translational Gastroenterology Unit, Nuffield Department of Medicine, University of Oxford, Oxford, UK

**Keywords:** Molecular biology, Immunology

## Abstract

In vitro-transcribed (IVT) mRNAs are modalities that can combat human disease, exemplified by their use as vaccines for severe acute respiratory syndrome coronavirus 2 (SARS-CoV-2). IVT mRNAs are transfected into target cells, where they are translated into recombinant protein, and the biological activity or immunogenicity of the encoded protein exerts an intended therapeutic effect^1,2^. Modified ribonucleotides are commonly incorporated into therapeutic IVT mRNAs to decrease their innate immunogenicity^3–5^, but their effects on mRNA translation fidelity have not been fully explored. Here we demonstrate that incorporation of *N*^1^-methylpseudouridine into mRNA results in +1 ribosomal frameshifting in vitro and that cellular immunity in mice and humans to +1 frameshifted products from BNT162b2 vaccine mRNA translation occurs after vaccination. The +1 ribosome frameshifting observed is probably a consequence of *N*^1^-methylpseudouridine-induced ribosome stalling during IVT mRNA translation, with frameshifting occurring at ribosome slippery sequences. However, we demonstrate that synonymous targeting of such slippery sequences provides an effective strategy to reduce the production of frameshifted products. Overall, these data increase our understanding of how modified ribonucleotides affect the fidelity of mRNA translation, and although there are no adverse outcomes reported from mistranslation of mRNA-based SARS-CoV-2 vaccines in humans, these data highlight potential off-target effects for future mRNA-based therapeutics and demonstrate the requirement for sequence optimization.

## Abstract (web summary)

A study demonstrates that nucleotide modifications in mRNA-based therapeutics can lead to +1 ribosomal frameshifting during translation, yielding products that can trigger immune responses.

## Main

A key feature of therapeutic IVT mRNAs is that they contain modified ribonucleotides, which have been shown to decrease innate immunogenicity and can additionally increase mRNA stability, both of which are favourable characteristics for mRNA therapies^1–5^. For example, clinically approved SARS-CoV-2 mRNA vaccines incorporate *N*^1^-methylpseudouridine (1-methylΨ), which has been shown to decrease IVT mRNA innate immunogenicity^3–5^. Some modified ribonucleotides, such as 5-methylcytidine (5-methylC), are naturally occurring post-transcriptional mRNA modifications in eukaryotes, whereas others are not, such as 1-methylΨ (refs. ^6–10^).

We investigated how 5-methoxyuridine (5-methoxyU), 5-methylC and 1-methylΨ affect translation of IVT mRNA. 5-methoxyU, 5-methylC and 1-methylΨ have been utilized in IVT mRNAs to attempt to increase recombinant protein synthesis in vitro, and for preclinical proof of concept for IVT mRNA-based therapies^11,12^. As mentioned, 1-methylΨ is a ribonucleotide incorporated in licensed IVT mRNA-based SARS-CoV-2 vaccines, but also mRNA-based human vaccines and therapies in development^2,13,14^.

Despite their widespread use, surprisingly little is known about how ribonucleotide modification affects protein synthesis, particularly for translation of therapeutic IVT mRNAs. We were interested in how modified ribonucleotides affect the fidelity of mRNA translation for several reasons. Certain ribonucleotide modifications can recode mRNA sequences (for example, inosine^15^). 5-methylC has previously been shown to increase misreading during mRNA translation in prokaryotes, but its effect on eukaryotic mRNA translation fidelity has not been explored^16^. The effect of 5-methoxyU on translation fidelity has not been investigated. Pseudouridine (Ψ) is known to increase misreading of mRNA stop codons in eukaryotes, and can affect misreading during prokaryotic mRNA translation^16–18^. 1-methylΨ does not seem to affect codon misreading, but has been shown to affect protein synthesis rates and ribosome density on mRNAs, suggesting a direct effect on mRNA translation^19,20^.

At present, it is unclear which modified ribonucleotides affect mRNA translation fidelity and existing studies are mostly limited to understanding misreading frequencies only at a given codon. Misreading of mRNA codons is also only one type of post-transcriptional mechanism that can alter a polypeptide sequence. So far, no study has investigated the fundamental question of whether modified ribonucleotides can affect the maintenance of the correct reading frame during translation of a synthetic transcript. Understanding these processes is critical to increase our knowledge of protein synthesis from modified mRNAs in general, but is also imperative for the robust design and evaluation of new mRNA-based therapeutics that make use of modified ribonucleotides within widely differing RNA sequences or therapeutic contexts.

**Fig. 1:**
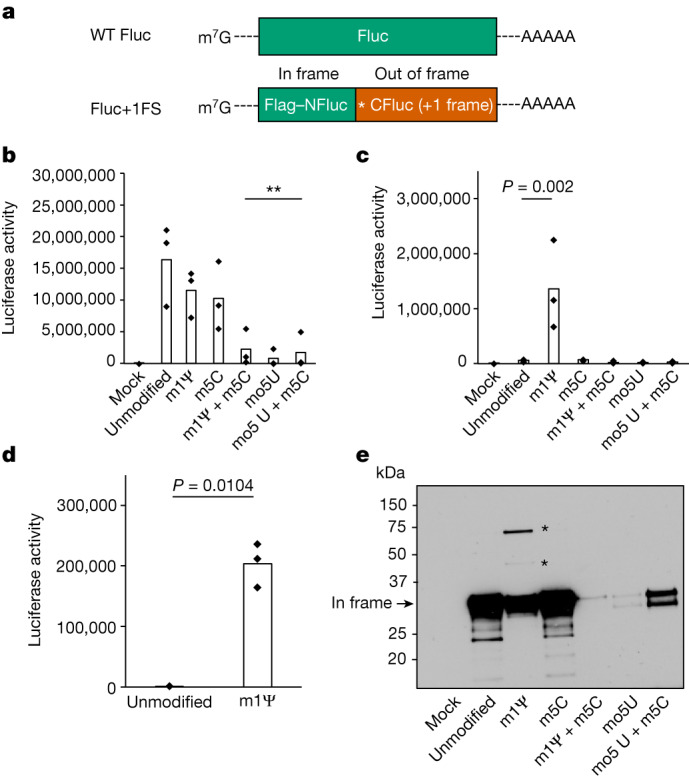
Translation of 1-methylΨ-modified mRNA produces +1 frameshifted polypeptides. **a**, Structures of IVT mRNA transcripts used to probe protein synthesis fidelity. WT Fluc contains only (in-frame) Fluc coding sequence. For Fluc+1FS, the green segment represents in-frame N-terminal Fluc coding sequence (NFluc), and the orange segment represents +1 frameshifted C-terminal Fluc coding sequence (CFluc). Asterisk represents a premature stop codon. **b**, Luciferase activity produced by translation of WT Fluc mRNAs, either unmodified control (canonical nucleotides), or containing 1-methylΨ (m1Ψ), 5-methylC (m5C), 5-methoxyU (mo5U) or the combinations indicated. **P < 0.01 (1-methylΨ + 5-methylC, *P* = 0.0051; 5-methoxyU, *P* = 0.0023; 5-methoxyU + 5-methylC, *P* = 0.0042; one-way analysis of variance (ANOVA) with Dunnett’s test). **c**, Luciferase activity produced by translation of modified Fluc+1FS mRNAs and unmodified control. 1-methylΨ, *P* = 0.002 (one-way ANOVA with Dunnett’s test). **d**, Luciferase activity in lysates produced by transfection of HeLa cells with unmodified or 1-methylΨ Fluc+1FS mRNA for 8 h. *P* = 0.0104 (Welch’s one-tailed *t*-test). **e**, Western blot analysis (anti-Flag epitope) of polypeptides produced by translation of mRNAs in **c**. All data are obtained from *n* = 3 replicated experiments. **e** shows a single blot from *n* = 3 replicated experiments. Asterisks represent bands at higher molecular weight. For gel source data, see Supplementary Fig. 2.

To investigate how ribonucleotide modification affects reading frame maintenance during translation of mRNA, we designed and synthesized IVT mRNAs (Fluc+1FS) that report on out-of-frame protein synthesis (Fig. 1a). Fluc+1FS mRNAs encode an amino-terminal segment of firefly luciferase (NFluc) and a complementary carboxy-terminal segment of Fluc (CFluc), directly downstream. CFluc is encoded in the +1 reading frame. Fluc+1FS mRNAs are designed to produce catalytically inactive (truncated) NFluc when translated normally. However, if ribosomes move out of frame during translation, elongated polypeptides containing residues from both in-frame NFluc and out-of-frame CFluc can be produced, which can increase catalytic activity.

**Extended Data Fig. 1:**
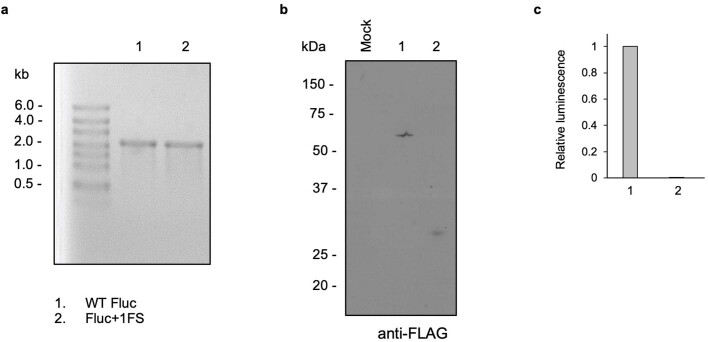
Validation of WT Fluc, Fluc + 1FS, and Fluc-1FS mRNAs. **a**, UV photograph of WTFluc and Fluc+1FS mRNA transcripts analysed by agarose gel electrophoresis. **b**, Immunoblot of luciferase protein produced by translation of WTFluc and Fluc+1FS mRNAs using anti-FLAG antibody. **c**, Relative luciferase expression produced by translation of WTFluc and Fluc+1FS mRNAs. Data were obtained from *n* = 1 (non-replicated) analyses. For gel source data, see Supplementary Fig. 6.

We synthesized unmodified Fluc+1FS mRNAs, which contain canonical ribonucleotides, and translated them in vitro. We confirmed that Fluc+1FS mRNAs produce catalytically inactive NFluc (Extended Data Fig. 1). By comparison, unmodified wild-type (WT) Fluc mRNA, containing the complete in-frame Fluc coding sequence, produced the expected active protein (Extended Data Fig. 1). Then we synthesized and translated each mRNA containing 5-methoxyU, 5-methylC, 1-methylΨ, 5-methoxyU + 5-methylC or 1-methylΨ + 5-methylC. Translation of WT Fluc mRNA was not significantly affected by either 1-methylΨ or 5-methylC modifications alone, but was decreased by incorporating both ribonucleotides into a single transcript (Fig. 1b). 5-methoxyU incorporation alone, or combined with 5-methylC, significantly decreased translation of WT Fluc mRNA (Fig. 1b). Incorporation of 1-methylΨ in Fluc+1FS mRNA significantly increased ribosomal +1 frameshifting to about 8% of the corresponding in-frame protein, which was not observed for other ribonucleotides (Fig. 1c). HeLa cells transfected with 1-methylΨ Fluc+1FS mRNA recapitulated the results from in vitro translation (Fig. 1d). On the basis of these observations, we concluded that IVT mRNA containing 1-methylΨ or 5-methylC exhibits similar translation efficiency to unmodified mRNA, but 1-methylΨ significantly increases ribosomal +1 frameshifting during mRNA translation.

We observed a large increase in ribosomal +1 frameshifting during translation of 1-methylΨ mRNA and reasoned that gaining better understanding of the translation products would complement the reporter assay data and help to explain how +1 frameshifted products originate. To address these aspects, we probed the polypeptides produced during IVT mRNA translation by western blotting. Translation of unmodified Fluc+1FS mRNA produced the expected in-frame truncated product, which was also true for 5-methylC mRNA (Fig. 1e). Translation of 1-methylΨ mRNA produced the expected in-frame product, but also produced two additional bands at higher molecular weight (Fig. 1e). We reasoned that these products were +1 frameshifted polypeptides. We also confirmed that 1-methylΨ + 5-methylC-, 5-methoxyU- and 5-methoxyU + 5-methylC mRNAs were comparatively poor mRNA templates for protein synthesis (Fig. 1e).

**Fig. 2:**
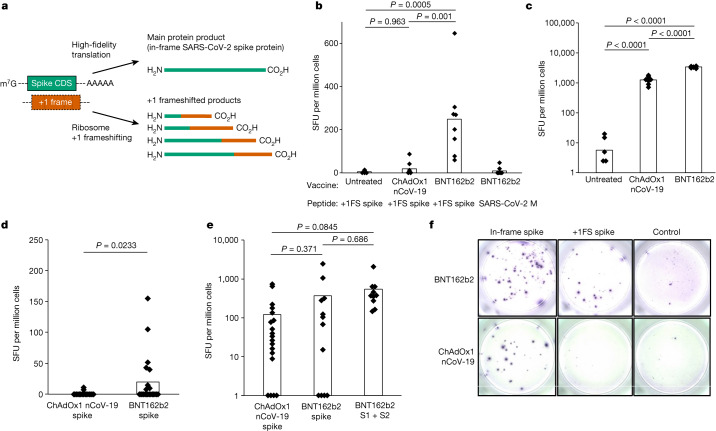
+1 frameshifted products elicit off-target cellular immune responses following modified mRNA vaccination. **a**, Depiction of spike and +1 frameshifted (+1FS) products produced by 1-methylΨ-modified spike mRNA translation. CDS, coding sequence. **b**, Splenocyte IFNγ ELISpot responses from untreated, ChAdOx1 nCoV-19-vaccinated or BNT162b2-vaccinated mice stimulated with +1FS spike peptides. IFNγ ELISpot response from BNT162b2-vaccinated mice stimulated with SARS-CoV-2 M peptides (unrelated control antigen) is included for additional comparison. SFU, spot-forming units. Each group *n* = 8. Untreated versus ChAdOx1 nCoV-19, *P* = 0.963; untreated versus BNT162b2, *P* = 0.0005; ChAdOx1 nCoV-19 versus BNT162b2, *P* = 0.001. **c**, Splenocyte IFNγ ELISpot responses from mice in **b** stimulated with spike peptides. Untreated versus ChAdOx1 nCoV-19, *P* = 2.05 × 10^−9^; untreated versus BNT162b2, *P* = 4.5 × 10^−14^; ChAdOx1 nCoV-19 versus BNT162b2, *P* = 1.88 × 10^−13^. **d**, Peripheral blood mononuclear cells (PBMC) IFNγ ELISpot responses from donors vaccinated with ChAdOx1 nCoV-19 (*n* = 20) or BNT162b2 (*n* = 21) stimulated with +1FS spike peptides. *P* = 0.0233 (Welch’s one-tailed *t*-test). **e**, PBMC IFNγ ELISpot responses from donors in **c** stimulated with in-frame spike peptides: total spike pool or spike S1 + S2 subpools. ChAdOx1 nCoV-19 spike versus BNT162b2 spike, *P* = 0.371; ChAdOx1 nCoV-19 spike versus BNT162b2 S1 + S2, *P* = 0.0845; BNT162b2 spike versus BNT162b2 S1 + S2, *P* = 0.686. **f**, Representative images of PBMC IFNγ ELISpot response wells for two individuals vaccinated with either BNT162b2 responder (top) or ChAdOx1 nCoV-19 (bottom). Left to right: in-frame spike response (spike peptides); +1FS spike response (+1FS spike peptides); no peptide control. *P* values in **b**,**c**,**e** were determined by one-way ANOVA and Tukey’s test. Source Data

1-methylΨ is also used in clinically approved SARS-CoV-2 mRNA vaccines^3,4^. As 1-methylΨ increased +1 ribosome frameshifting during translation in vitro, we investigated whether this occurs in vivo for BNT162b2, a SARS-CoV-2 mRNA vaccine containing 1-methylΨ. We reasoned that +1 ribosomal frameshifting during recombinant antigen mRNA translation could lead to presentation of +1 frameshifted products to T cells, and elicit off-target cellular immune responses (Fig. 2a). Antigen presentation from mistranslation of endogenous tumour mRNA has been shown to occur in vivo (for example, ref. ^21^). To address this possibility, we vaccinated mice with BNT162b2 and quantified their T cell response to in-frame SARS-CoV-2 spike protein and +1 frameshifted products predicted to occur by translation of the mRNA +1 frame, as well as an unrelated control antigen (SARS-CoV-2 M protein), by interferon-γ (IFNγ) ELISpot assay. Junction peptides consisting of in-frame N-terminal residues and C-terminal +1 frameshifted residues were not included. We found that responses to +1 frameshifted spike peptides were significantly increased in vaccinated mice compared to untreated mice or those vaccinated with ChAdOx nCoV-19, which does not produce antigen from translation of *N*^1^-methylpseudouridylated mRNA^22^ (Fig. 2b). Both BNT162b2 and ChAdOx1 nCoV-19 vaccination produced ELISpot responses to in-frame SARS-CoV-2 spike (Fig. 2c). These data suggest that +1 frameshifted products encoded in BNT162b2 spike mRNA are T cell antigens for inbred mice, to which off-target immunity can be detected following vaccination.

**Extended Data Fig. 2:**
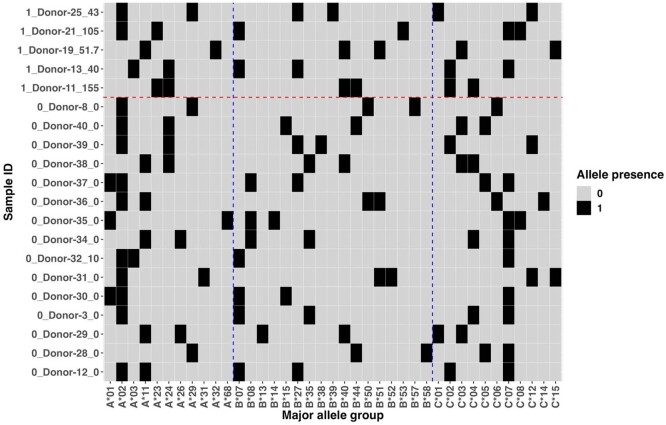
Visualisation of HLA genotypes in BNT162b2-vaccinated individuals. Major allele groups in HLA-A, -B and -C genes for *n* = 40 donors. The Sample ID contains 3 fields separated by underline: (i) 1 or 0, with 1 denoting an ELISpot count >40 following stimulation with the +1 FS peptide pool; (ii) donor identifier; (iii) ELISpot count if >40. SFU/million. Allele group frequency indicated by black tile, vertical dashed lines: HLA genes, horizontal dashed line: indicates the cut-off (ELISpot count >40).

**Extended Data Fig. 3:**
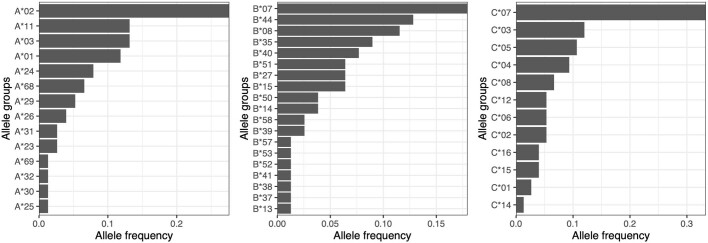
HLA allele frequency distributions. Frequencies of each major allele group in HLA-A, -B and -C genes for *n* = 40 donors.

**Extended Data Fig. 4:**
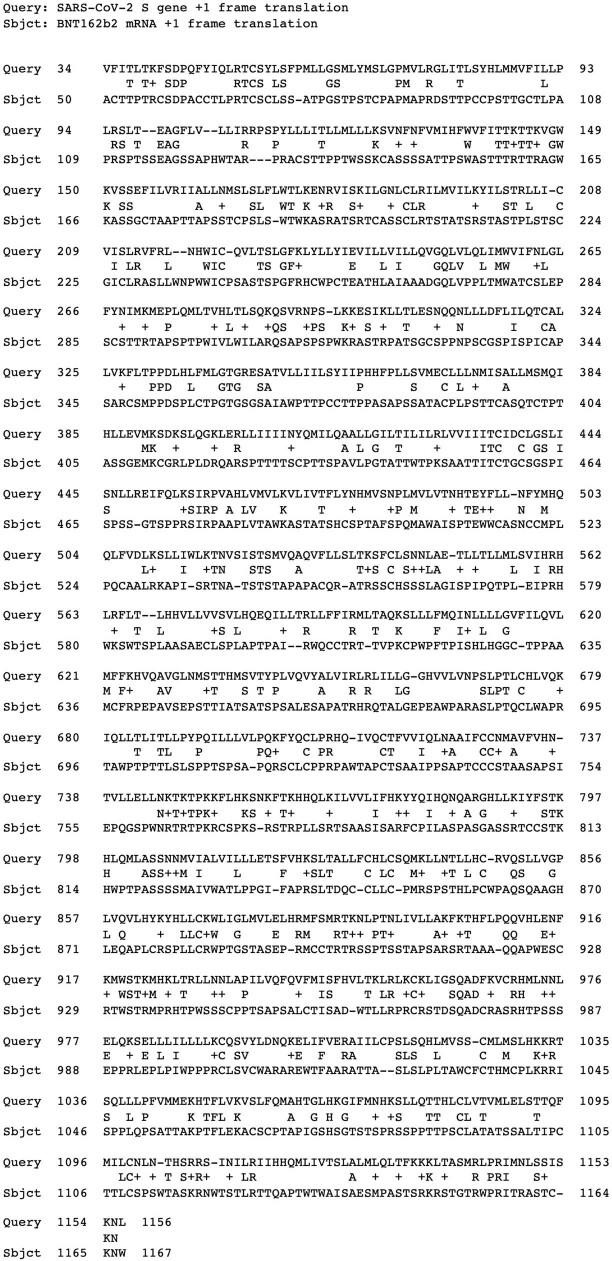
NCBI BLASTP alignment of + 1 translated products. Protein BLAST alignment of polypeptides predicted by +1 frame translation of either BNT162b2 mRNA or Wuhan SARS-CoV-2 Spike mRNA (from NC_045512.2).

We then compared IFNγ ELISpot responses to predicted +1 frameshifted SARS-CoV-2 spike protein products in 21 individuals vaccinated with BNT162b2 and compared these responses to those of 20 individuals vaccinated with ChAdOx1 nCoV-19, none of whom reported undue effects as a result of vaccination. We detected a significantly higher IFNγ response to +1 frameshifted antigen in the BNT162b2 vaccine group, compared to ChAdOx1 nCoV-19 (Fig. 2d). There was no association between T cell responses to +1 frameshifted antigen and age, sex or HLA subtype (Supplementary Table 1 and Extended Data Figs. 2 and 3). Both ChAdOx1 nCoV-19 and BNT162b2 vaccination produced ELISpot responses to in-frame SARS-CoV-2 spike, but responses to +1 frameshifted products were observed only in individuals vaccinated with BNT162b2 (Fig. 2e,f). During SARS-CoV-2 viral replication, a programmed −1 ribosomal frameshift occurs naturally during translation of open reading frame (ORF) 1a and ORF1b (ref. ^23^). It is not feasible that these data are a consequence of natural SARS-CoV-2 infection for the following, non-exhaustive, reasons. First, no frameshifting activity is known to occur during SARS-CoV-2 spike subgenomic mRNA translation (which would be a major discovery in its own right). Second, −1 frameshifting (and not +1 frameshifting) is restricted to a single programmed site in ORF1a and ORF1b (ref. ^23^). Third, +1 frameshifted peptides are predicted from the BNT162b2 mRNA sequence, and not the S gene sequence from wild virus (Extended Data Fig. 4). Instead, these data suggest that vaccination with 1-methylΨ mRNA can elicit cellular immunity to peptide antigens produced by +1 ribosomal frameshifting in both major histocompatibility complex (MHC)-diverse people and MHC-uniform mice.

**Fig. 3:**
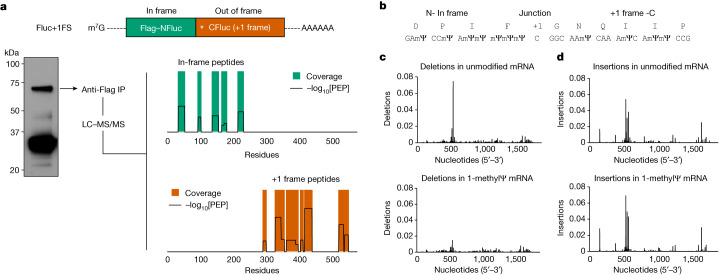
Mistranslation of 1-methylΨ mRNA is due to +1 ribosomal frameshifting and not transcriptional errors. **a**, Tryptic peptide coverage plot of the purified high molecular weight polypeptide produced by translation of 1-methylΨ Fluc+1FS mRNA, showing in-frame residues (top) and +1 frameshifted residues (bottom). −log_10_[PEP] is the mass spectrum percolator score (only high-quality peptides are shown). IP, immunoprecipitate. The structure of Fluc+1FS mRNA from Fig. 1 is re-displayed and a western blot of the translation reaction before immunoprecipitation is displayed. For gel source data, see Supplementary Fig. 3. **b**, Junction peptide derived from +1 ribosomal frameshifting and the originating mRNA sequence. **c**, Nucleotide deletions in unmodified (top) and 1-methylΨ (bottom) Fluc+1FS mRNA, quantified by *n* = 3 RNA-sequencing analyses. **d**, Nucleotide insertions in unmodified (top) and 1-methylΨ (bottom) Fluc+1FS mRNA.

**Extended Data Fig. 5:**
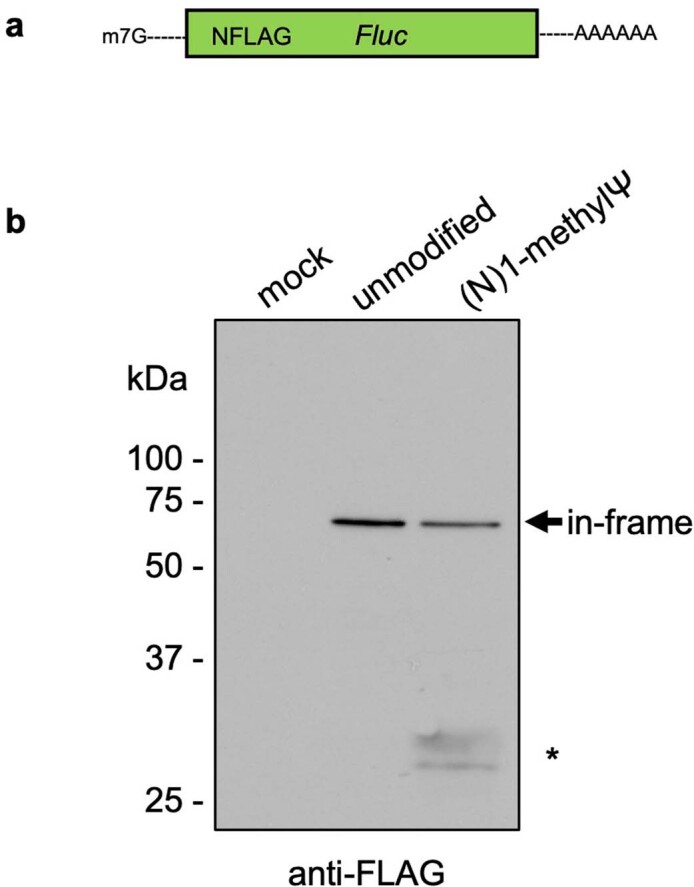
Translation of 1-methylΨ-modified Fluc mRNA produces multiple polypeptides. **a**, Diagram of NFLAG-WTFluc mRNA. **b**, Western blot analysis (anti-FLAG epitope) of polypeptides produced by translation of unmodified, or 1-methylΨ, NFLAG-WTFluc mRNA. In-frame firefly luciferase is indicated by arrow. Low molecular weight polypeptides are indicated by asterisk (*). A single blot from *n* = 3 replicated experiments is displayed. For gel source data, see Supplementary Fig. 7.

To provide further mechanistic insight into +1 ribosome frameshifting during translation of 1-methylΨ mRNA, and identify potential frameshift sites or sequences, we translated 1-methylΨ Fluc+1FS mRNA, purified the major putative +1 frameshifted polypeptide and carried out liquid chromatography tandem mass spectrometry (LC–MS/MS) of tryptic digests. From this single polypeptide, we identified six in-frame peptides and nine peptides derived from the mRNA +1 frame (Fig. 3a and Extended Data Table 1). All in-frame peptides were mapped to the N-terminal region, whereas +1 frameshifted peptides were mapped downstream (Fig. 3a). We then repeated this analysis using a different protease and identified a junction peptide spanning the main frame and the +1 frame (Fig. 3b). These data demonstrated that the elongated polypeptide was indeed a chimeric polypeptide consisting of in-frame N-terminal residues and +1 frameshifted C-terminal residues. As expected, shorter frameshifted products were also produced from translation of 1-methylΨ mRNA encoding full-length Fluc (Extended Data Fig. 5).

Apparent errors in protein synthesis, including frameshifting, can be consequences of DNA mutation or transcriptional errors^24^. Hence, faithful translation of an incorrect mRNA sequence can produce incorrect proteins. In vitro transcripts are presumed to be exact RNA copies of template DNA, the accuracy of which may be estimated by the fidelity of the used RNA polymerase. However, the substitution of canonical substrate ribonucleoside triphosphates for modified nucleotides may increase transcriptional errors. To address this possibility, we carried out high-throughput RNA sequencing of unmodified and 1-methylΨ Fluc+1FS mRNA and quantified nucleotide insertions and deletions in each population of IVT mRNA. Nucleotide deletion profiles for each mRNA were very similar (Fig. 3c), as were nucleotide insertions (Fig. 3d), suggesting few site-specific differences. The overall frequency of insertions and deletions was low, and did not differ significantly between unmodified and 1-methylΨ mRNA (Extended Data Table 2), which is supported by recent observations^25^. From these findings, we concluded that frameshifted products of 1-methylΨ mRNA translation were not due to transcriptional errors, but were due to bona fide ribosomal +1 frameshifting—a post-transcriptional mechanism.

**Fig. 4:**
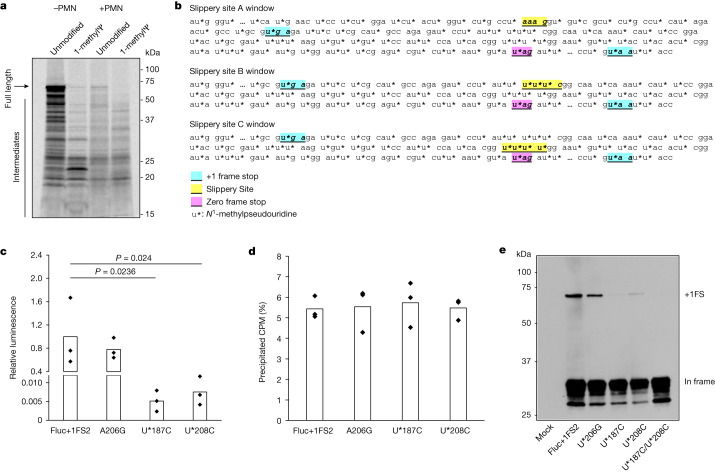
+1 ribosomal frameshifting is dependent on mRNA slippery sequences and associated with ribosome stalling during 1-methylΨ mRNA translation. **a**, SDS–polyacrylamide gel electrophoresis autoradiograph of [^35^S]methionine-labelled polypeptides produced by translation of unmodified or 1-methylΨ Fluc mRNA for 30 min, including or omitting 100 μM paromomycin (+PMN and −PMN, respectively). **b**, Diagram showing putative mRNA slippery sequences and stop-codon-flanked windows. **c**, Activity of +1 frameshifted products after translation of 1-methylΨ mutant mRNAs, or 1-methylΨ Fluc+1FS2 control mRNA, for 2 h. Fluc+1FS2 versus U*187C, *P* = 0.024; Fluc+1FS2 versus U*208C, *P* = 0.0236 (one-way ANOVA with Dunnett’s test). **d**, Total mRNA translation over 2 h for each of Fluc+1FS2 mRNA or mutant mRNAs, quantified by [^35^S]methionine incorporation. CPM, counts per minute. **e**, Western blot analysis (anti-Flag epitope) of polypeptides produced by translation of mRNAs in **c**, and U*187C/U*208C double-mutant 1-methylΨ mRNA. Data are from *n* = 3 replicated experiments. **a** and **e** show representative images from *n* = 3 replicated experiments. For gel source data, see Supplementary Figs. 4 and 5.

Ribosome frameshifting is a well-documented phenomenon that occurs during translation of many naturally occurring mRNAs^24^. As ribosome stalling is implicated in several instances of +1 frameshifting, we queried how the presence of 1-methylΨ in IVT mRNA affects translation elongation^26–28^. To do this, we assayed protein synthesis during translation of unmodified or 1-methylΨ WT Fluc mRNA using co-translational [^35^S]methionine labelling^29^. Translation elongation of 1-methylΨ mRNA was slower than for unmodified mRNA (Fig. 4a), which is supported by previous observations^20^. All reactions were run for 30 min and there was less full-length protein produced from the translation of 1-methylΨ-containing mRNAs, suggesting a slower elongation rate compared to that of unmodified mRNA, with a greater proportion of premature polypeptide products. These data suggested that elongating ribosomes stall during translation of mRNA containing 1-methylΨ.

It was unclear whether 1-methylΨ affected mRNA decoding rates, or another process, during elongation. We reasoned that slower decoding of 1-methylΨ codons during translation elongation could lead to ribosome stalling, similar to previous observations for ‘hungry’ codons at sites of +1 frameshifting during translation of naturally occurring mRNA^21,28^. We probed the molecular mechanism of ribosome stalling during 1-methylΨ mRNA translation using the aminoglycoside paromomycin. In brief, during mRNA decoding, cognate aminoacyl-tRNA anticodon–codon interaction causes local conformational changes in 18S rRNA (in eukaryotes), after which a new peptide bond is formed, ribosome subunit rotation occurs, and subsequent ribosome conformational changes, elongation factor 2 binding and translocation to the next codon completes the elongation cycle^30^. Paromomycin binds to helix 44 of 18S rRNA in elongating ribosomes and alters its conformation in the decoding centre, which inhibits translation but also permits the productive binding of near- and non-cognate aminoacyl-tRNAs to the 80S ribosome A-site^31^. In doing so, paromomycin increases the misincorporation of amino acids into elongating polypeptides^32^. We reasoned that if slow decoding during 1-methylΨ mRNA translation was due to altered aminoacyl-tRNA binding kinetics, this process could be decreased by paromomycin. This is because paromomycin-bound ribosomes could incorporate additional near- or non-cognate aminoacyl-tRNAs and effectively increase the substrate aminoacyl-tRNA pool at ribosome stall sites. Translation of 1-methylΨ mRNA was slower than that of unmodified mRNA and the proportion of premature polypeptide products was greater (Fig. 4a). However, during 1-methylΨ mRNA translation, polypeptide elongation was improved by the addition of paromomycin, whereas paromomycin was inhibitory only to unmodified mRNA translation (Fig. 4a). Together, these data show that slow translation of 1-methylΨ mRNA is probably due to ribosome stalling, which is caused by altered aminoacyl-tRNA binding, and which can be rescued by increasing the incorporation of near- or non-cognate amino acids into elongating polypeptides.

**Extended Data Fig. 6:**
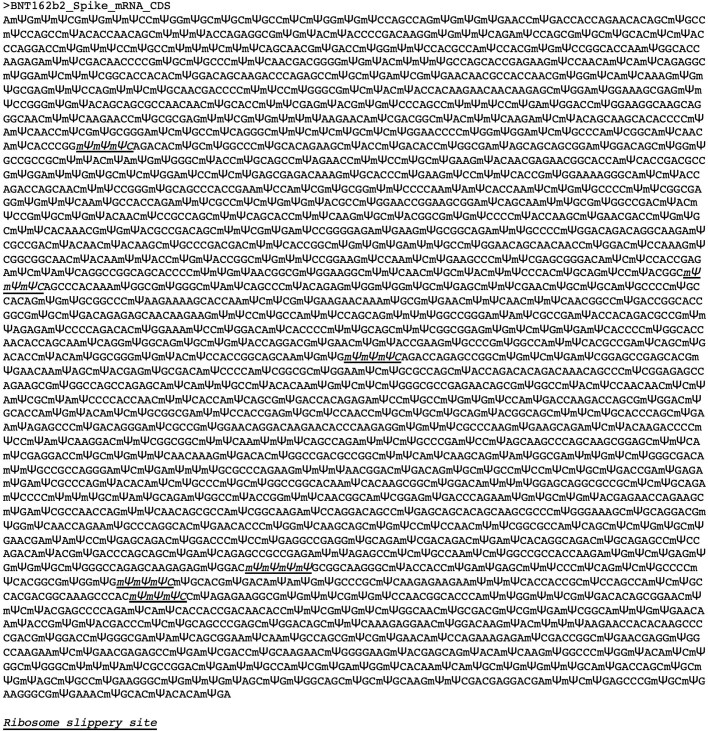
Annotated BNT162b2 Spike mRNA CDS putative slippery sites. Putative ribosome slippery sites in BNT162b2 which were identified by the following formula: m1Ψm1Ψm1Ψ X, where m1Ψ is (N)1-methylpseudouridine and X is (N)1-methylpseudouridine or cytidine in the first nucleotide of the immediate downstream codon.

Although there is no evidence that frameshifted products in humans generated from BNT162b2 vaccination are associated with adverse outcomes, for future use of mRNA technology it is important that mRNA sequence design is modified to reduce ribosome frameshifting events, as this may limit its future use for applications that require higher doses or more frequent dosing, such as the in vivo production of hormones. It is important to continue investigating therapeutic mRNA mistranslation and immunogenicity, as the evolution of antibody and cytolytic T cell responses against +1 frameshifted spike variants and peptides has not been systematically evaluated in humans and ELISpot responses obtained from pooled peptides may also underestimate T cell responses. The main in-frame mRNA-encoded product is unlikely to elicit an adaptive immune response, but presentation of +1 frameshifted products could activate T cells that target host cells. We reasoned that if we were able to identify +1 ribosome frameshift sites or sequences it would be possible to alter the mRNA sequence to reduce such effects. As proof of principle, we used our reporter IVT mRNA system. LC–MS/MS analysis showed that translation of 1-methylΨ mRNA leads to synthesis of +1 frameshifted products within the area of coding sequence between detected in-frame residues and downstream +1 frameshifted residues (Fig. 3a). We searched the RNA sequence corresponding to this region in the junction peptide coding sequence (Fig. 3b) and determinants of ribosome frameshifting from published mechanisms, from which we identified three potential ribosome slippery sequences (Fig. 4c), with all three sequences having the potential to be decoded by the same aminoacyl-tRNA at an in-frame codon or in the immediate +1 frame codon. Notably, six slippery sites identical to Fluc+1FS slippery sites B and C were also distributed in the BNT162b2 spike mRNA coding sequence. These sites have been annotated in the Fluc+1FS coding sequence (Fig. 4b) and the BNT163b2 spike mRNA coding sequence (Extended Data Fig. 6). We reasoned that these sequences could therefore function as sites for +1 ribosomal frameshifting. We synonymously mutated each site in 1-methylΨ Fluc+1FS mRNA such that the in-frame amino acid was unchanged, but the immediate +1 frame codon was mutated to a non-cognate amino acid, hence destroying the ribosome slippery sequence, and translated the mRNAs to evaluate the contribution of each site to +1 ribosomal frameshifting (Fig. 4c). A +1 frame stop codon was present downstream of slippery site A, and it was unlikely that frameshifting at this site contributed to increased luciferase activity. As expected, luciferase activity produced by translation of site A mutant A206G mRNA was the same as control levels (Fig. 4c). However, both slippery site B mutant U*187C mRNA and slippery site C mutant U*208C mRNA strongly decreased +1 ribosome frameshifting (Fig. 4c). Notably, translation efficiency of each mRNA was equal, which suggests that no mutation adversely affected mRNA translation overall, but solely +1 ribosomal frameshifting activity (Fig. 4d). Translation of a U*187C/U*208C double-mutant 1-methylΨ Fluc+1FS mRNA produced no detectable +1 ribosome frameshifting (Fig. 4e). The transframe protein product predicted by +1 frameshifting at slippery site C contains an alteration of 19 amino acid residues (compared to WT Fluc), whereas +1 frameshifting at slippery site B produces a transframe product that is effectively 100% homologous to WT Fluc. In addition, given that mutation of either slippery site B or C (U*187C or U*208C) significantly decreased luciferase activity, but that relatively more frameshifted product was produced by translation of U*208C mRNA (Fig. 4e), we reasoned that the transframe product produced by frameshifting at slippery site C had lower specific luciferase activity, and that frameshifting at slippery site B contributed to most of the detected luciferase activity as a consequence of +1 ribosome frameshifting. Taken together, these data suggest that *N*^1^-methylpseudouridylation at defined mRNA sequences triggers ribosome +1 frameshifting; however, with appropriate mRNA sequence design, it is possible to ameliorate this issue.

## Conclusions

We show that 1-methylΨ is a modified ribonucleotide that significantly increases +1 ribosomal frameshifting during mRNA translation and that cellular immunity to +1 frameshifted products can occur following vaccination with mRNA containing 1-methylΨ. To our knowledge, this is the first report that mRNA modification affects ribosomal frameshifting. Alongside this impact on host T cell immunity, the off-target effects of ribosomal frameshifting could include increased production of new B cell antigens. Other ribonucleotide modification strategies, such as incorporation of 5-methoxyU, significantly decreased translation efficiency of IVT mRNAs, which may limit clinical translation. Although we have shown that translation of *N*^1^-methylpseudouridylated mRNA leads to +1 ribosomal frameshifting in vitro and in cultured cells, it is conceivable that other mistranslation events (such as leaky scanning) could also contribute to T cell responses to +1 frameshifted peptide antigens. We show that IVT mRNAs contain few nucleotide insertions and deletions, and this is not changed by 1-methylΨ incorporation. Our data show that +1 ribosomal frameshifting occurs at two characterized slippery sequences. Therefore, we believe that the minor band of approximately 50 kDa produced by Fluc+1FS mRNA translation is probably a consequence of several frameshifting events (Fig. 1e). Translation of mRNA containing 1-methylΨ leads to slower translation elongation, caused by altered aminoacyl-tRNA binding, which demonstrates why +1 ribosomal frameshifting does not occur during unmodified mRNA translation—both ribosome stalling and ribosome slippery sequences seem to be required for productive +1 ribosome frameshifting. Our mechanistic data are supported by previous observations of ribosomal frameshifting during translation of naturally occurring mRNAs, which implicate ribosome stalling and require ribosome slippery sequences for +1 frameshifting^21,26–28,33,34^. These findings are of particular importance to our fundamental understanding of how ribonucleotide modification affects mRNA translation, and for designing and optimizing future mRNA-based therapeutics to avoid mistranslation events that may decrease efficacy or increase toxicity.

## Methods

### Plasmids and mRNA synthesis

Phusion High-Fidelity DNA polymerase reagents were obtained from New England Biolabs. In-frame WT Fluc template DNA was produced by XbaI digest of pUCK100Fluc, including an 80-nucleotide polyA tail^29^. Fluc+1FS template DNA was produced by overlap extension PCR of pUCK100Fluc using FlucFlag_F (5′-TTATACCATGGGTGACTACAAAGACCATGACGGTGATTATAAAGATCATGACATCGATTACAAGGATGACGATGACAAGCTCGAAGACGCCAAAAACATAAAGAAAGG-3′), Fluc+1FS_R (5′-GATTGCCGAAAAATAGGATCTCTGGCATG-3′) for Fluc+1FS NFluc, Fluc+1FS_F (5′-CAGAGATCCTATTTTTCGGCAATCAAATCAT-3′) and Fluc_R (5′-TAGATTGCTAGCTTATGTTAATTACACGGCGATCTTTCCG-3′) for Fluc+1FS CFluc. PCR products were reinserted into pUCK100 using NcoI and NheI, and linear template DNA was produced by XbaI digest. A206G, U*187C and U*208C mRNA were transcribed from custom genes subcloned into pUC57T7 (Genscript Biotech) and linear template DNA was produced by BamHI digest. Fluc+1FS2 mRNA was produced from Fluc+1FS template DNA subcloned into pUC57T7 and linearized by BamHI. U*187C/U*208C template DNA was produced by overlap extension PCR and reinsertion into pUC57. Reporter RNA sequences are shown in Supplementary Fig. 1. In vitro transcription was carried out using TranscriptAid T7 High Yield Transcription Kit (Thermo Scientific K0441). UTP and CTP were substituted where required for 5-methoxyUTP, *N*^1^-methylpseudoUTP or 5-methylCTP. Modified nucleotides were obtained from Trilink Biotechnologies. Transcripts were 5′-capped using the Vaccinia Capping System (NEB M2080S) and purified by phenol–chloroform extraction and G50 size exclusion. Transcripts were quantified using a Nanodrop ND2000 spectrophotometer (Thermo Scientific) and stored at −80 °C.

### RNA gel electrophoresis

Samples were heated in formamide with bromophenol blue and xylene cyanol dye for 3 min at 95 °C, cooled for 2 min on ice and resolved on a 1% agarose formaldehyde MOPS acetate gel for 90 min at 90 V. The gel was stained in 0.5 µg ml^−1^ ethidium bromide for 1 h, bathed in distilled water for 1 h and visualized by UV transillumination.

### Cell culture and mRNA transfection

HeLa cells were a gift from the Proudfoot Lab, University of Oxford. Cells were authenticated by STR typing. Cells were grown in DMEM (Gibco 41966029), supplemented with 10% FBS at 37 °C, 5% CO_2_. Cells were tested for mycoplasma contamination and tested negative. Approximately 16 h before transfection, cells were seeded at 0.2 × 10^6^ ml^−1^ in 6-well plates. Ten minutes before transfection, the medium was changed to OptiMEM (Gibco 31985062), after which cells were transfected with 4 pmol Fluc+1FS mRNA in Lipofectamine 2000 (Invitrogen 11668019). After 4 h transfection, OptiMEM was replaced with DMEM, and cells were cultured for a further 4 h, and then lysed in Passive Lysis Buffer (Promega E1941). Lysates were centrifuged (10,000*g*, 5 min) and luciferase activity was determined in supernatants using the Luciferase Assay System (Promega E4550) and GloMax multi-well plate luminometer (Promega).

### In vitro translation

IVT mRNAs were translated using the Flexi Rabbit Reticulocyte Lysate System using nuclease-treated RRL (Promega L4540). For co-translational labelling, 0.33 µl translation-grade [^35^S]methionine (Hartman Analytic KSM-01) and 0.67 µl amino acids minus methionine (Promega L996A) were used per 15 µl reaction. Unlabelled products were produced with 1 µl total (unlabelled) amino acids (Promega L4461). The concentration of IVT mRNA was 50 nM and, when included, the concentration of paromomycin (Sigma Aldrich P9297) was 100 µM. Creatine phosphate (Roche 10621714001), creatine kinase (Roche 21778721), potassium acetate (Sigma Aldrich P1190) and magnesium acetate (Sigma Aldrich M5661) were included at 10 mM, 25 µg ml^−1^, 50 mM and 0.5 mM, respectively^35^. Reactions were incubated at 30 °C for the indicated time and moved to ice, after which 10 µl RNase A/T1 and Benzonase was added and incubated for 10 min. Luciferase activity was determined using the Luciferase Assay System (Promega E4550) and measured using a GloMax multi-well plate luminometer (Promega). The relative proportion of gain-of-function luciferase activity from Fluc+1FS mRNA translation was calculated by the relative light units (RLUs) for each mRNA translation reaction as a percentage of unmodified WT Fluc mRNA translation RLUs. For western blotting, 2× reducing LDS PAGE buffer was mixed with each sample, which was heated to 70 °C for 10 min. Cooled samples were resolved on NuPAGE 4–12% or 12%, Bis-Tris, 1.0 mm, Mini Protein Gels (Invitrogen). The resolved products were transferred to nitrocellulose membrane and probed using anti-Flag M2 antibody (Sigma Aldrich F1804) and anti-mouse-HRP antibody (Dako P0447), and detected with Clarity Western ECL substrate (Bio-Rad 1705060). Figures 1 and 4 show translation reactions from *n* = 3 replicates.

### Peptide LC–MS/MS analysis

IVT mRNA was translated as above. After RNA digestion, translation products were immunoprecipitated using anti-Flag magnetic agarose beads (Pierce) overnight at 4 °C. Beads were washed twice in PBS, once in water, eluted in LDS PAGE buffer, and resolved on a NuPAGE 4–12%, Bis-Tris, 1.5 mm, Mini Protein Gel (NP0335BOX). The gel was stained with Coomassie dye and the region between about 60 kDa and 75 kDa (Precision Plus Protein All Blue Prestained Protein Standard; Bio-Rad) was excised and processed for mass spectrometry analysis as previously described^36^. In brief, the excised gel slice was cut into 1-mm pieces and placed in an 1.5-ml microtube. Coomassie staining was removed by incubating alternatively with a mixture of 25 mM ammonium bicarbonate and acetonitrile (2:1) and 25 mM ammonium bicarbonate. Each 15-min incubation at 37 °C was repeated until gel pieces were completely destained. Reduction and alkylation of cysteines was carried out by first incubating with a fresh 10 mM final concentration of dithiothreitol in 25 mM ammonium bicarbonate at 60 °C for 60 min and then changing the solution to 60 mM final concentration of iodoacetamide in 25 mM ammonium bicarbonate and incubating for an addition 45 min at room temperature in the dark. After dehydrating the gel pieces with acetonitrile, trypsin solution (10 ng μl^−1^ in 25 mM ammonium bicarbonate) or AspN (1 ng μl^−1^ in 25 mM ammonium bicarbonate) was added until gel pieces were completely covered. Digestion was carried at 37 °C for 16 h, 1,000 r.p.m. shaking. Proteases were inactivated by adding formic acid (trypsin digest) or TFA (AspN digest) to a final concentration of 1% (v/v). Peptides were extracted by sequential incubations with water/acetonitrile/formic acid (50:49:1 and 80:19:1% (v/v)). Extracted peptides were pooled and dried to completion and resuspended in water/acetonitrile (97:3% (v/v)) with 0.1% (v/v) TFA for mass spectrometry analysis. Mass spectrometry analysis was carried out once for trypsin digest and once for AspN digest.

### Mass spectrometry analysis

In-gel digests were analysed using an Ultimate 3000 RSLC nano system (Thermo Scientific) coupled to an Orbitrap Eclipse mass spectrometer (Thermo Scientific). The sample was loaded onto the trapping column (Thermo Scientific, PepMap100, C18, 300 μm × 5 mm), using partial loop injection, for 3 min at a flow rate of 15 μl min^−1^ with 0.1% (v/v) FA in 3% acetonitrile. Peptides were separated on the analytical column (Easy-Spray C18 75 µm × 500 mm 2 µm column) at a flow rate of 300 nl min^−1^ using a gradient of 97% A (0.1% formic acid)/3% B (80% acetonitrile 0.1% formic acid) to 25% B over 50 min, then to 40% B for an additional 6 min, and then to 90% B for another 2 min, remaining at 90% B for 12 min before the percentage of B was then lowered to 3.8% to allow the column to re-equilibrate for 15 min before the next injection. Data were acquired using two field asymmetric ion mobility spectrometry CVs (−50 V, −70 V). For each field asymmetric ion mobility spectrometry experiment (maximum cycle time of 1.5 s per experiment), data were acquired in data-dependent mode and MS1 consisted of a 120,000 resolution full-scan MS scan (AGC set to 100% (4 × 10^5^ ions) with a maximum fill time of 50 ms) using a mass range of 380–1,500 *m*/*z*. The intensity MS2 trigger threshold was set to 5.0 × 10^3^, and to avoid repeated selection of peptides for MS/MS, the experiment used a 40-s dynamic exclusion window. MS/MS was carried out on the Orbitrap using 30,000 resolution (AGC set to 100% (5 × 10^4^ ions) with a maximum fill time of 54 ms). A higher-energy collisional dissociation collision energy of 32% was used to fragment the peptides and an isolation window of 1.2 was used.

### Proteome Discoverer v2.5 analysis

Raw data were imported and data were processed in Proteome Discoverer (version 2.5, Thermo Fisher Scientific). The raw files were submitted to a database search using Proteome Discoverer with SequestHF against the *Oryctolagus cuniculus* (rabbit) database containing protein sequences from UniProt/Swiss-Prot (Proteome ID UP000001811), appended with Fluc (Uniprot ID P08659), predicted +1 frameshifted polypeptides and common contaminant proteins (several types of human keratin, BSA and porcine trypsin). The spectrum identification was carried out with the following parameters: MS accuracy, 10 ppm; MS/MS accuracy of 0.02 Da; up to two missed cleavage sites allowed; carbamidomethylation of cysteine; and oxidation of methionine as variable modifications. An interactive workflow was used in the processing step. After the first Sequest HT search, the Inferis Rescoring node was used and spectra with confidence worse than high were resubmitted for a second Sequest HT search using additional dynamic modifications (N and Q deamidation; N-terminal pyroglutamate; methionine loss and acetylation). Peptides were assigned to their respective reading frame or junction by inspection. Percolator node was used for false discovery rate estimation and only rank 1 peptide identifications of high confidence (false discovery rate < 1%) were accepted.

### RNA-sequencing analysis

RNA-sequencing (RNA-seq) libraries were prepared from 1 µg IVT mRNA using NextFlex Rapid Directional RNA-seq kit 2.0 (PerkinElmer), according to the manufacturer’s protocol. Libraries were amplified by six PCR cycles and purified by PAGE. Sequencing was carried out using an Illumina MiSeq at the Department of Biochemistry DNA sequencing facility, University of Cambridge (1 × 150 cycles V3). Reads were aligned with STAR (version 2.7.4a)^37^. Insertions and deletions per reference nucleotide were mapped from high-quality reads (QC score > 35) filtered for partial alignments and normalized to read depth. Insertion or deletion plots show the average mutation frequency for *n* = 3 replicated RNA-seq experiments.

### SDS–PAGE autoradiography

IVT mRNAs were translated in nuclease-treated RRL (Promega) and products were co-translationally labelled as described above for 30 min. 2× LDS PAGE buffer was mixed to each sample, which was heated to 70 °C for 10 min. Cooled samples were resolved on NuPAGE 12%, Bis-Tris, 1.0 mm, Mini Protein Gels (Invitrogen NP0342BOX). The resolved gels were fixed in 10% methanol in acetic acid for 45 min, and dried at 80 °C for 2 h using a Fisher gel dryer system. Images were obtained by autoradiography using a Typhoon FLA 9000 and storage phosphor screens (GE Healthcare).

### Incorporated [^35^S]methionine quantification

IVT mRNAs were translated in nuclease-treated RRL (Promega) and products were co-translationally labelled for 2 h, as described above. [^35^S]methionine incorporation was assayed according to the manufacturer’s protocol. In brief, after RNA digestion, reactions were incubated for 10 min in 1 M NaOH. Polypeptides were precipitated in 5% TCA, collected on Whatman glass fibre filters, and washed three times with 5% TCA and once with acetone. The dried filters were immersed in 2 ml EcoScint liquid scintillation cocktail (National Diagnostics) and counted in a Tri-Carb 4910 TR liquid scintillation counter (PerkinElmer). Incorporated [^35^S]methionine was determined from cpm of precipitated polypeptides per counts per minute of unwashed filters for each reaction (total counts per minute).

### Mouse immunization

C57BL/6J mice (female, WT) were purchased from Charles Rivers Laboratories. Mice of 8–12 weeks old were intramuscularly injected with three doses of 10 μg BNT162b2, 5 × 10^7^ infectious units of ChAdOx1 nCoV-19 or untreated. For booster immunizations, the same dose of the respective vaccine was injected 3 weeks and 6 weeks apart into the same site as the primary immunization. Spleens were obtained at day 8 post-third dose and cell suspensions were prepared. In brief, spleens were mashed with a syringe plunge and filtered through 70-μm cell strainers. Red blood cells were lysed with RBC lysing buffer (155 mM NH_4_Cl, 12 mM NaHCO_3_, 0.1 mM EDTA), before counting and cryopreserving before ELISpot assays. Mice were not randomly assigned to groups and experimenters were not blinded to experiments.

### IFNγ ELISpot

Human IFNγ ELISpot assays were carried out as previously described using the human IFNγ ELISpot PLUS kit (ALP; MabTech 3420-4APT)^38^. Overlapping peptide pools corresponding to: in-frame SARS-CoV-2 spike protein (spike, 158 peptides); spike protein S1 and S2 regions (S1 + S2), which were described previously^38^; SARS-CoV-2 M protein, which was described previously^38^; and peptides predicted to occur by translation of the BNT162b2 mRNA +1 frame exclusively (+1FS spike, 123 peptides) were used. Peptides were obtained from Mimotopes, and are listed in Extended Data Table 3. Cryopreserved PBMCs were thawed in RPMI1640 medium supplemented with 1% (v/v) penicillin–streptomycin (Sigma), containing 0.01% (v/v) Benzonase nuclease (Merck). PBMCs were washed and then incubated for 1–2 h at 37 °C, 5% CO_2_ in RPMI1640 medium, 10% (v/v) human AB serum and 1% (v/v) penicillin–streptomycin. Pre-coated IFNγ ELISpot 96-well plates (MabTech 3420-4APT-2) were washed three times with PBS and then blocked with RPMI1640 medium, 10% (v/v) human AB serum and 1% (v/v) penicillin–streptomycin for 45 min. Overlapping peptide pools were plated at 4 µg ml^−1^, 50 µl per well; dimethylsulfoxide (Sigma) was used as the negative control at the equivalent concentration to the peptides. A total of 200,000 cells in 50 µl were added and incubated for 18–24 h. Cells were discarded, and plates were washed with PBS 0.05% (v/v) Tween (Sigma), and incubated with IFNγ detector antibody (clone 7-B6-1, 1 µg ml^−1^) for 2–4 h at room temperature. Washed plates were then incubated with streptavidin alkaline phosphatase antibody (1 µg ml^−1^) for 1–2 h. Plates were washed and then colour development was carried out using 1-step NBT/BCIP substrate solution. A 50 µl volume of filtered NBT/BCIP was added to each well for 5 min at room temperature after which development was stopped with cold water. Plates were dried at room temperature for approximately 48 h. Spots were quantified using an AID iSpot Spectrum EliSpot Reader (AID EliSpot Software version 7.0, Autoimmun Diagnostika). Average spot count value in the background wells was subtracted from that of the test wells and values were expressed as SFUs per million cells. Mouse IFNγ ELISpot assays were carried out using cryopreserved splenocytes thawed as above and incubated in RPMI1640 medium with 10% FBS alone. Peptide stimulations and downstream processing were as above, using pre-coated mouse IFNγ ELISpot PLUS kit (ALP; MabTech 3321-4APT-2). Figure 2b,c shows ELISpot responses for *n* = 8 mice per group. Figure 2d,e shows ELISpot responses for *n* = 20 ChAdOx1 nCoV-19-vaccinated individuals and *n* = 21 BNT162b2-vaccinated individuals.

### HLA genotyping

HLA genotyping was conducted for *n* = 40 donors by Histogenetics LLC (Ossining, New York). HLA data (Extended Data Table 1) were filtered to *HLA-A*, *HLA-B* and *HLA-C* genes and were truncated to allele group level. Donor genotypes for the BNT162b2-vaccinated individuals were visualized in a presence–absence heatmap in R (version 4.3.0) using ggplot2 (version 3.4.2). Major allele group information was summarized and individual allele group frequencies were calculated to illustrate the overall genetic composition.

### Statistical analysis

Statistical analyses were carried out in base R (version 4.3.0) or using the DescTools R package (version 0.99.46).

### Ethics statement

Animal experiments were licensed by the UK Home Office according to the Animals Scientific Procedures Act 1986 (License PP6047951), and approved and conducted in compliance with protocols by the University of Cambridge, University Biomedical Services Animal Welfare and Ethical Review Bodies committee. Human sample collection and analysis was conducted in accordance with the principles of good clinical practice and following approved protocols of the NIHR National BioResource. Samples were collected with the written informed consent of all study participants under the NIHR National BioResource-Research Tissue Bank ethics (research ethics committee (REC): 17/EE/0025) and from the PITCH study. PITCH is a substudy of the SIREN study, which was approved by the Berkshire REC, Health Research 250 Authority (IRAS ID 284460, REC reference 20/SC/0230), with PITCH recognized as a substudy on 2 December 2020. SIREN is registered with ISRCTN (Trial ID: 252 ISRCTN11041050). Some participants were recruited under aligned study protocols. In Liverpool, some participants were recruited under the study Human immune responses to acute virus infections (16/NW/0170), approved by North West - Liverpool Central REC on 8 March 2016, and amended on 14 September 2020 and 4 May 2021. In Oxford, participants were recruited under the GI Biobank Study 16/YH/0247, approved by the REC at Yorkshire & The Humber - Sheffield REC on 29 July 2016, which was amended for this purpose on 8 June 2020. The study was conducted in compliance with all relevant ethical regulations for work with human participants, and according to the principles of the Declaration of Helsinki (2008) and the International Conference on Harmonization Good Clinical Practice guidelines. Written informed consent to publish clinical and genetic data, as well as for study participation, was obtained for all participants enrolled in the study.

### Reporting summary

Further information on research design is available in the Nature Portfolio Reporting Summary linked to this article.

## Online content

Any methods, additional references, Nature Portfolio reporting summaries, source data, extended data, supplementary information, acknowledgements, peer review information; details of author contributions and competing interests; and statements of data and code availability are available at 10.1038/s41586-023-06800-3.

### Supplementary information

Supplementary Figures Supplementary Figs. 1–7, displaying reporter RNA sequences and uncropped films/gel photographs/autoradiograph scans.

Reporting Summary

Supplementary Table 1 Participant age, sex, HLA genotype, vaccine modality and ELISpot +1FS spike response.

### Source data

Source Data Fig. 2

## Figures and Tables

**Extended Data Table 1:**
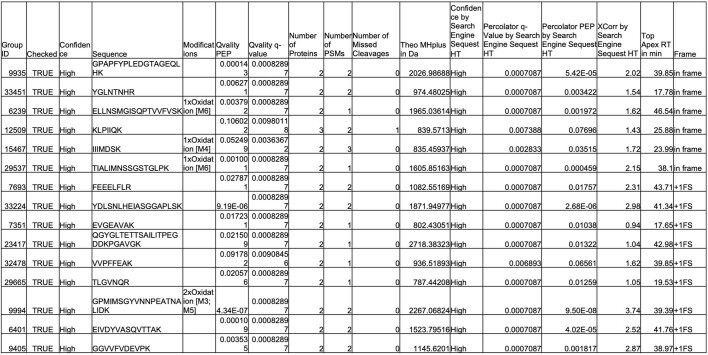
LC-MS/MS analysis of Fluc + 1FS high-molecular weight polypeptide

Tryptic peptides identified by purification and LC-MS/MS analysis of the high molecular weight product produced by 1-methylΨ Fluc+1FS mRNA translation.

**Extended Data Table 2:**
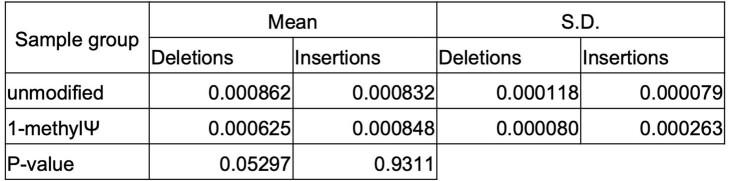
Insertion and Deletion Frequencies in unmodified and 1-methylΨ mRNAs

Comparison of single nucleotide deletion frequency or insertion frequency, normalised to total reads (%), for unmodified or 1-methylΨ mRNA (Welch’s unpaired two-tailed T-test).

**Extended Data Table 3:**
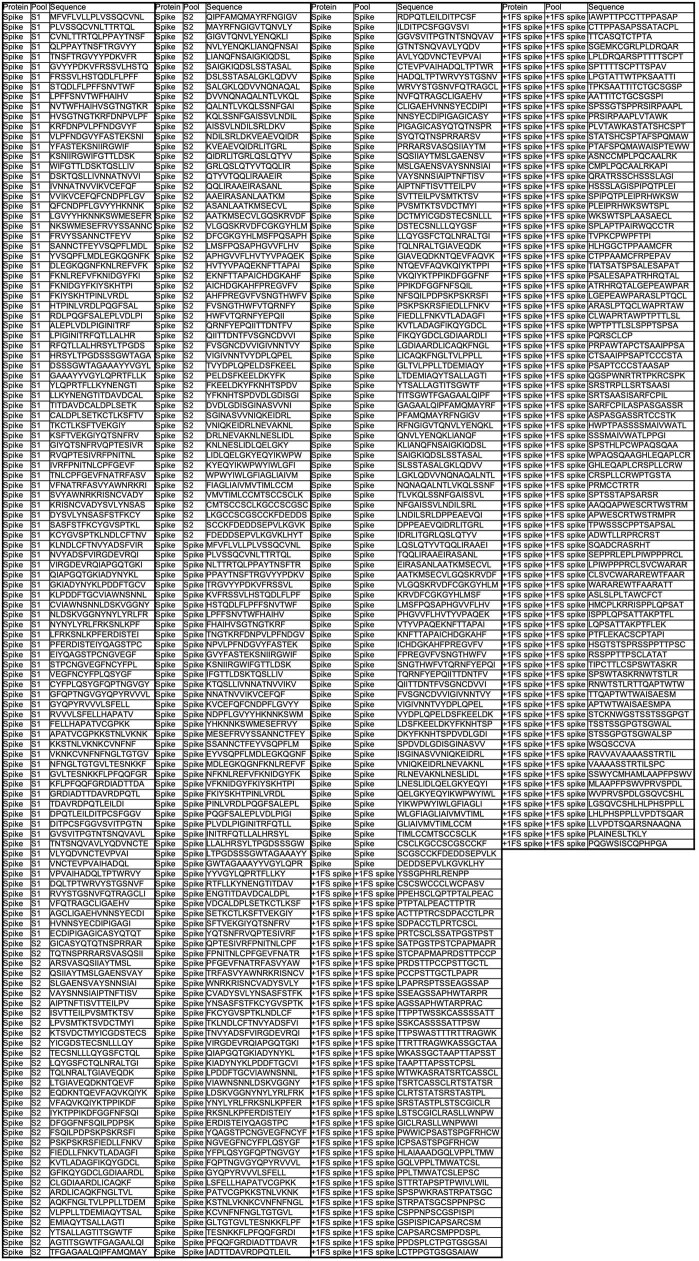
Spike and + 1FS Spike peptides

List of peptides in each pool used for Spike and +1FS Spike ELISpot analysis.

## Data Availability

Mass spectrometry data have been deposited with MassIVE ID MSV000093074. RNA-seq reads and processed files are available at the NCBI Gene Expression Omnibus (accession GSE223044). Additional data are available from figshare (10.6084/m9.figshare.24271744). The following accessions were used for mass spectrometry analysis: UP000001811 and P08659 (UniProt). Source data are provided with this paper.

## Extended data

Extended data is available for this paper at 10.1038/s41586-023-06800-3.

## Code availability

Scripts for processing alignments are available from GitHub^39^.

## References

[1] Hogan MJ, Pardi N (2022). mRNA vaccines in the COVID-19 pandemic and beyond. Annu. Rev. Med..

[2] Chaudhary N, Weissman D, Whitehead KA (2021). mRNA vaccines for infectious diseases: principles, delivery and clinical translation. Nat. Rev. Drug Discov..

[3] Anderson BR (2010). Incorporation of pseudouridine into mRNA enhances translation by diminishing PKR activation. Nucleic Acids Res..

[4] Holtkamp S (2006). Modification of antigen-encoding RNA increases stability, translational efficacy, and T-cell stimulatory capacity of dendritic cells. Blood.

[5] Andries O (2015). N^1^-methylpseudouridine-incorporated mRNA outperforms pseudouridine-incorporated mRNA by providing enhanced protein expression and reduced immunogenicity in mammalian cell lines and mice. J. Control. Release.

[6] Boo SH, Kim YK (2020). The emerging role of RNA modifications in the regulation of mRNA stability. Exp. Mol. Med..

[7] Squires JE (2012). Widespread occurrence of 5-methylcytosine in human coding and non-coding RNA. Nucleic Acids Res..

[8] Argoudelis AD, Mizsak SA (1976). 1-methylpseudouridine, a metabolite of *Streptomyces platensis*. J. Antibiot..

[9] Pang H (1982). Structure of a modified nucleoside in archaebacterial tRNA which replaces ribosylthymine. 1-Methylpseudouridine. J. Biol. Chem..

[10] Brand RC, Klootwijk J, Planta RJ, Maden BE (1978). Biosynthesis of a hypermodified nucleotide in *Saccharomyces carlsbergensis* 17S and HeLa-cell 18S ribosomal ribonucleic acid. Biochem. J..

[11] Li B, Luo X, Dong Y (2016). Effects of chemically modified messenger RNA on protein expression. Bioconjugate Chem..

[12] Zangi L (2013). Modified mRNA directs the fate of heart progenitor cells and induces vascular regeneration after myocardial infarction. Nat. Biotechnol..

[13] Stadler CR (2017). Elimination of large tumors in mice by mRNA-encoded bispecific antibodies. Nat. Med..

[14] Pardi N (2018). Nucleoside-modified mRNA immunization elicits influenza virus hemagglutinin stalk-specific antibodies. Nat. Commun..

[15] Licht K (2019). Inosine induces context-dependent recoding and translational stalling. Nucleic Acids Res..

[16] Hoernes TP (2016). Nucleotide modifications within bacterial messenger RNAs regulate their translation and are able to rewire the genetic code. Nucleic Acids Res..

[17] Karijolich J, Yu YT (2011). Converting nonsense codons into sense codons by targeted pseudouridylation. Nature.

[18] Eyler DE (2019). Pseudouridinylation of mRNA coding sequences alters translation. Proc. Natl Acad. Sci. USA.

[19] Kim KQ (2022). N1-methylpseudouridine found within COVID-19 mRNA vaccines produces faithful protein products. Cell Rep..

[20] Svitkin YV (2017). N1-methyl-pseudouridine in mRNA enhances translation through eIF2α-dependent and independent mechanisms by increasing ribosome density. Nucleic Acids Res..

[21] Bartok O (2021). Anti-tumour immunity induces aberrant peptide presentation in melanoma. Nature.

[22] Folegatti PM (2020). Safety and immunogenicity of the ChAdOx1 nCoV-19 vaccine against SARS-CoV-2: a preliminary report of a phase 1/2, single-blind, randomised controlled trial. Lancet.

[23] Bhatt PR (2021). Structural basis of ribosomal frameshifting during translation of the SARS-CoV-2 RNA genome. Science.

[24] Champagne J, Mordente K, Nagel R, Agami R (2022). Slippy-Sloppy translation: a tale of programmed and induced-ribosomal frameshifting. Trends Genet..

[25] Chen TH, Potapov V, Dai N, Ong JL, Roy B (2022). *N*^1^-methyl-pseudouridine is incorporated with higher fidelity than pseudouridine in synthetic RNAs. Sci. Rep..

[26] Simms CL, Yan LL, Qiu JK, Zaher HS (2019). Ribosome collisions result in +1 frameshifting in the absence of no-go decay. Cell Rep..

[27] Juszkiewicz S, Hegde RS (2017). Initiation of quality control during poly(A) translation requires site-specific ribosome ubiquitination. Mol. Cell.

[28] O’Connor M (2002). Imbalance of tRNA(Pro) isoacceptors induces +1 frameshifting at near-cognate codons. Nucleic Acids Res..

[29] Stoneley M (2022). Unresolved stalled ribosome complexes restrict cell-cycle progression after genotoxic stress. Mol. Cell.

[30] Lareau LF, Hite DH, Hogan GJ, Brown PO (2014). Distinct stages of the translation elongation cycle revealed by sequencing ribosome-protected mRNA fragments. Elife.

[31] Prokhorova I (2017). Aminoglycoside interactions and impacts on the eukaryotic ribosome. Proc. Natl Acad. Sci. USA.

[32] Tuite MF, McLaughlin CS (1984). The effects of paromomycin on the fidelity of translation in a yeast cell-free system. Biochim. Biophys. Acta.

[33] Jacks T, Madhani HD, Masiarz FR, Varmus HE (1988). Signals for ribosomal frameshifting in the Rous sarcoma virus gag-pol region. Cell.

[34] Devaraj A, Fredrick K (2010). Short spacing between the Shine-Dalgarno sequence and P codon destabilizes codon-anticodon pairing in the P site to promote +1 programmed frameshifting. Mol. Microbiol..

[35] Svitkin Y. V. & Sonenberg N. in *mRNA Processing and Metabolism* Vol. 257 (eds Schoenberg, D. R.) 155–170 (Humana, 2004).

[36] Rosenfeld J, Capdevielle J, Guillemot JC, Ferrara P (1992). In-gel digestion of proteins for internal sequence analysis after one- or two-dimensional gel electrophoresis. Anal. Biochem..

[37] Dobin A (2013). STAR: ultrafast universal RNA-seq aligner. Bioinformatics.

[38] Payne RP (2021). Immunogenicity of standard and extended dosing intervals of BNT162b2 mRNA vaccine. Cell.

[39] Mulroney, T. E. RNA-seq_mutations. GitHub https://github.com/tom-mulroney/rna-seq_mutations (accessed 23 January 2023).

