# Supplementary material for: N1-methylpseudouridylation of mRNA causes +1 ribosomal frameshifting
Source: Nature. 2023 Dec 6;625(7993):189–94. doi: 10.1038/s41586-023-06800-3 (PMC10764286; doi:10.1038/s41586-023-06800-3)
Supplement: Supplementary file 1 — Supplementary Figs. 1–7, displaying reporter RNA sequences and uncropped films/gel photographs/autoradiograph scans. [file 41586_2023_6800_MOESM1_ESM.pdf]

nature portfolio

https://doi.org/10.1038/s41586-023-06800-3

Supplementary information

N^1-methylpseudouridylation of mRNA causes +1 ribosomal frameshifting

In the format provided by the
authors and unedited

Supplementary Information

(N)1-methylpseudouridylation of mRNA causes +1 ribosomal frameshifting

Thomas E. Mulroney[1], Tuija Pöyry[1], Juan Carlos Yam-Puc[1], Maria Rust[1], Robert F Harvey[1], Lajos Kalmar[1], Emily Horner[1], Lucy Booth[1], Alexander P. Ferreira[1], Mark Stoneley[1], Ritwick Sawarkar[1], Alexander J. Mentzer[2], Kathryn S Lilley[3], C. M. Smales[4,10], Tobias Von der Haar[4], Lance Turtle[5], Susanna Dunachie[6,7,8], Paul Klenerman[6,9] James E. D. Thaventhiran[1,11*], and Anne E. Willis,[1,11*].

[1] MRC Toxicology Unit, Gleeson Building, Tennis Court Road, University of Cambridge, CB2 1QR

[2] Welcome Centre for Human Genetics, University of Oxford, Old Road Campus, Oxford,

[3] Department of Biochemistry, University of Cambridge, Tennis Court Rd, Cambridge CB2 1QW

[4] School of Biosciences, Division of Natural Sciences, University of Kent, Canterbury, CT2 7NJ, UK

[5]NIHR Health Protection Research Unit for Emerging and Zoonotic Infections, Institute of Infection, Veterinary and Ecological Sciences, University of Liverpool, Liverpool, UK.

[6]NIHR Oxford Biomedical Research Centre, Oxford University Hospitals NHS Foundation Trust, Oxford, UK

[7]NDM Centre for Global Health Research, Nuffield Department of Medicine, University of Oxford, Oxford, UK.

[8]Mahidol-Oxford Tropical Medicine Research Unit, Mahidol University, Bangkok, Thailand

[9]Translational Gastroenterology Unit, Nuffield Department of Medicine, University of Oxford, Oxford, UK.

[10]National Institute for Bioprocessing Research and Training, University College Dublin, Foster Avenue, Mount Merrion, Dublin, Ireland

[11]These authors contributed equally

*Correspondence: James Thaventhiran (jedt2@cam.ac.uk); Anne Willis

(aew80@cam.ac.uk).

Supplementary Information Table of Contents

Supplementary Fig. 1: Reporter RNA sequences.
Supplementary Fig. 2: Uncropped films for western blot displayed in Figure 1e.
Supplementary Fig. 3. Uncropped film for western blot displayed in Figure 3a.
Supplementary Fig. 4: Uncropped autoradiograph scans for autoradiograph displayed in Figure 4a.
Supplementary Fig. 5: Uncropped films for western blot displayed in Figure 4e.
Supplementary Fig. 6. Uncropped images for Extended Data Fig. 1.
Supplementary Fig. 7: Uncropped films for western blot displayed in Figure 4e.
Supplementary Table 1: Participant age, sex, HLA genotype, vaccine modality, and ELISpot +1FS spike response.

>WTFluc

GGACU*AGU*AU*U*U*CAGU*U*GCAGU*AU*AAU*AU*AGU*U*AAGU*AGU*U*AGAAGU*AU*U*GU*AAU*AU*U*CCCAU*GGGU*CACCACCACCACCACCACGAAGACGCCAAAAACAU*AAAG
AAAGGCCCGGCGCCAU*U*CU*AU*CCGCU*GGAAGAU*GGAACCGCU*GGAGAGCAACU*GCAU*AAGGCU*AU*GAAGAGAU*ACGCCCU*GGU*U*CCU*GGAACAAU*U*GCU*U*U*U*ACAGAU*G
CACAU*AU*CGAGGU*GGACAU*CACU*U*ACGCU*GAGU*ACU*U*CGAAAU*GU*CCGU*U*CGGU*U*GGCAGAAGCU*AU*GAAACGAU*AU*GGGCU*GAAU*ACAAAU*CACAGAAU*CGU*CGU*
AU*GCAGU*GAAAACU*CU*CU*U*CAAU*U*CU*U*U*AU*GCCGGU*GU*U*GGGCGCGU*U*AU*U*U*AU*CGGAGU*U*GCAGU*U*GCGCCCGCGAACGACAU*U*U*AU*AAU*GAACGU*GAAU
*U*GCU*CAACAGU*AU*GGGCAU*U*U*CGCAGCCU*ACCGU*GGU*GU*U*CGU*U*U*CCAAAAAGGGGU*U*GCAAAAAAU*U*U*U*GAACGU*GCAAAAAAAGCU*CCCAAU*CAU*CCAAAAAAU
*U*AU*U*AU*CAU*GGAU*U*CU*AAAACGGAU*U*ACCAGGGAU*U*U*CAGU*CGAU*GU*ACACGU*U*CGU*CACAU*CU*CAU*CU*ACCU*CCCGGU*U*U*U*AAU*GAAU*ACGAU*U*U*U*
GU*GCCAGAGU*CCU*U*CGAU*AGGGACAAGACAAU*U*GCACU*GAU*CAU*GAACU*CCU*CU*GGAU*CU*ACU*GGU*CU*GCCU*AAAGGU*GU*CGCU*CU*GCCU*CAU*AGAACU*GCCU*GC
GU*GAGAU*U*CU*CGCAU*GCCAGAGAU*CCU*AU*U*U*U*U*GGCAAU*CAAAU*CAU*U*CCGGAU*ACU*GCGAU*U*U*U*AAGU*GU*U*GU*U*CCAU*U*CCAU*CACGGU*U*U*U*GGAAU
*GU*U*U*ACU*ACACU*CGGAU*AU*U*U*GAU*AU*GU*GGAU*U*U*CGAGU*CGU*CU*U*AAU*GU*AU*AGAU*U*U*GAAGAAGAGCU*GU*U*U*CU*GAGGAGCCU*U*CAGGAU*U*ACAAG
AU*U*CAAAGU*GCGCU*GCU*GGU*GCCAACCCU*AU*U*CU*CCU*U*CU*U*CGCCAAAAGCACU*CU*GAU*U*GACAAAU*ACGAU*U*U*AU*CU*AAU*U*U*ACACGAAAU*U*GCU*U*CU*G
GU*GGCGCU*CCCCU*CU*CU*AAGGAAGU*CGGGGAAGCGGU*U*GCCAAGAGGU*U*CCAU*CU*GCCAGGU*AU*CAGGCAAGGAU*AU*GGGCU*CACU*GAGACU*ACAU*CAGCU*AU*U*CU*GA
U*U*ACACCCGAGGGGGAU*GAU*AAACCGGGCGCGGU*CGGU*AAAGU*U*GU*U*CCAU*U*U*U*U*U*GAAGCGAAGGU*U*GU*GGAU*CU*GGAU*ACCGGGAAAACGCU*GGGCGU*U*AAU*CA
AAGAGGCGAACU*GU*GU*GU*GAGAGGU*CCU*AU*GAU*U*AU*GU*CCGGU*U*AU*GU*AAACAAU*CCGGAAGCGACCAACGCCU*U*GAU*U*GACAAGGAU*GGAU*GGCU*ACAU*U*CU*GGA
GACAU*AGCU*U*ACU*GGGACGAAGACGAACACU*U*CU*U*CAU*CGU*U*GACCGCCU*GAAGU*CU*CU*GAU*U*AAGU*ACAAAGGCU*AU*CAGGU*GGCU*CCCGCU*GAAU*U*GGAAU*CCA
U*CU*U*GCU*CCAACACCCCAACAU*CU*U*CGACGCAGGU*GU*CGCAGGU*CU*U*CCCGACGAU*GACGCCGGU*GAACU*U*CCCGCCGCCGU*U*GU*U*GU*U*U*U*GGAGCACGGAAAGACGA
U*GACGGAAAAAGAGAU*CGU*GGAU*U*ACGU*CGCCAGU*CAAGU*AACAACCGCGAAAAAGU*U*GCGCGGAGGAGU*U*GU*GU*U*U*GU*GGACGAAGU*ACCGAAAGGU*CU*U*ACCGGAAAAC
U*CGACGCAAGAAAAAU*CAGAGAGAU*CCU*CAU*AAAGGCCAAGAAGGGCGGAAAGAU*CGCCGU*GCU*CGACGAU*U*ACAAAGACGACGACGACAAGGGU*GACU*AU*AAAGACGACGAU*GAU*A
AAAU*U*GACU*ACAAGGAU*GACGAU*GACAAGU*AAGCU*AGCU*ACCCAU*ACGAU*GU*U*CCAGAU*U*ACGCU*CU*CGAGAAAAAAAAAAAAAAAAAAAAAAAAAAAAAAAAAAAAAAAAAAAAA
AAAAAAAAAAAAAAAAAAAAAAAAAAAAAAAAAAAAAGU*CU*U*

>Fluc+1FS

GGACU*AGU*AU*U*U*CAGU*U*GCAGU*AU*AAU*AU*AGU*U*AAGU*AGU*U*AGAAGU*AU*U*GU*AAU*AU*U*CCCAU*GGGU*GACU*ACAAAGACCAU*GACGGU*GAU*U*AU*AAAGAU*
CAU*GACAU*CGAU*U*ACAAGGAU*GACGAU*GACAAGCU*CGAAGACGCCAAAAACAU*AAAGAAAGGCCCGGCGCCAU*U*CU*AU*CCGCU*GGAAGAU*GGAACCGCU*GGAGAGCAACU*GCAU*A
AGGCU*AU*GAAGAGAU*ACGCCCU*GGU*U*CCU*GGAACAAU*U*GCU*U*U*U*ACAGAU*GCACAU*AU*CGAGGU*GGACAU*CACU*U*ACGCU*GAGU*ACU*U*CGAAAU*GU*CCGU*U*CGG
U*U*GGCAGAAGCU*AU*GAAACGAU*AU*GGGCU*GAAU*ACAAAU*CACAGAAU*CGU*CGU*AU*GCAGU*GAAAACU*CU*CU*U*CAAU*U*CU*U*U*AU*GCCGGU*GU*U*GGGCGCGU*U*AU
*U*U*AU*CGGAGU*U*GCAGU*U*GCGCCCGCGAACGACAU*U*U*AU*AAU*GAACGU*GAAU*U*GCU*CAACAGU*AU*GGGCAU*U*U*CGCAGCCU*ACCGU*GGU*GU*U*CGU*U*U*CCAAAA
AGGGGU*U*GCAAAAAAU*U*U*U*GAACGU*GCAAAAAAAGCU*CCCAAU*CAU*CCAAAAAAU*U*AU*U*AU*CAU*GGAU*U*CU*AAAACGGAU*U*ACCAGGGAU*U*U*CAGU*CGAU*GU*ACA
CGU*U*CGU*CACAU*CU*CAU*CU*ACCU*CCCGGU*U*U*U*AAU*GAAU*ACGAU*U*U*U*GU*GCCAGAGU*CCU*U*CGAU*AGGGACAAGACAAU*U*GCACU*GAU*CAU*GAACU*CCU*CU*
GGAU*CU*ACU*GGU*CU*GCCU*AAAGGU*GU*CGCU*CU*GCCU*CAU*AGAACU*GCCU*GCGU*GAGAU*U*CU*CGCAU*GCCAGAGAU*CCU*AU*U*U*U*U*CGGCAAU*CAAAU*CAU*U*CC
GGAU*ACU*GCGAU*U*U*U*AAGU*GU*U*GU*U*CCAU*U*CCAU*CACGGU*U*U*U*GGAAU*GU*U*U*ACU*ACACU*CGGAU*AU*U*U*GAU*AU*GU*GGAU*U*U*CGAGU*CGU*CU*U*A
AU*GU*AU*AGAU*U*U*GAAGAAGAGCU*GU*U*U*CU*GAGGAGCCU*U*CAGGAU*U*ACAAGAU*U*CAAAGU*GCGCU*GCU*GGU*GCCAACCCU*AU*U*CU*CCU*U*CU*U*CGCCAAAAGCA
CU*CU*GAU*U*GACAAAU*ACGAU*U*U*AU*CU*AAU*U*U*ACACGAAAU*U*GCU*U*CU*GGU*GGCGCU*CCCCU*CU*CU*AAGGAAGU*CGGGGAAGCGGU*U*GCCAAGAGGU*U*CCAU*CU
*GCCAGGU*AU*CAGGCAAGGAU*AU*GGGCU*CACU*GAGACU*ACAU*CAGCU*AU*U*CU*GAU*U*ACACCCGAGGGGGAU*GAU*AAACCGGGCGCGGU*CGGU*AAAGU*U*GU*U*CCAU*U*U*
U*U*U*GAAGCGAAGGU*U*GU*GGAU*CU*GGAU*ACCGGGAAAACGCU*GGGCGU*U*AAU*CAAAGAGGCGAACU*GU*GU*GU*GAGAGGU*CCU*AU*GAU*U*AU*GU*CCGGU*U*AU*GU*AAA
CAAU*CCGGAAGCGACCAACGCCU*U*GAU*U*GACAAGGAU*GGAU*GGCU*ACAU*U*CU*GGAGACAU*AGCU*U*ACU*GGGACGAAGACGAACACU*U*CU*U*CAU*CGU*U*GACCGCCU*GAAG
U*CU*CU*GAU*U*AAGU*ACAAAGGCU*AU*CAGGU*GGCU*CCCGCU*GAAU*U*GGAAU*CCAU*CU*U*GCU*CCAACACCCCAACAU*CU*U*CGACGCAGGU*GU*CGCAGGU*CU*U*CCCGACG
AU*GACGCCGGU*GAACU*U*CCCGCCGCCGU*U*GU*U*GU*U*U*U*GGAGCACGGAAAGACGAU*GACGGAAAAAGAGAU*CGU*GGAU*U*ACGU*CGCCAGU*CAAGU*AACAACCGCGAAAAAGU*
U*GCGCGGAGGAGU*U*GU*GU*U*U*GU*GGACGAAGU*ACCGAAAGGU*CU*U*ACCGGAAAACU*CGACGCAAGAAAAAU*CAGAGAGAU*CCU*CAU*AAAGGCCAAGAAGGGCGGAAAGAU*CGCCG
U*GU*AAU*U*AACAU*AAGCU*AGCU*ACCCAU*ACGAU*GU*U*CCAGAU*U*ACGCU*CU*CGAGAAAAAAAAAAAAAAAAAAAAAAAAAAAAAAAAAAAAAAAAAAAAAAAAAAAAAAAAAAAAAAA
AAAAAAAAAAAAAAAGU*CU*U*CU*

>FLAG-WTFluc

GGAAAU*AAGAGAGAAAAGAAGAGU*AAGAAGAAAU*AU*AACAGCCACCAU*GGGU*GACU*ACAAAGACCAU*GACGGU*GAU*U*AU*AAAGAU*CAU*GACAU*CGAU*U*ACAAGGAU*GACGAU*G
ACAAGCU*CGAAGACGCCAAAAACAU*AAAGAAAGGCCCGGCGCCAU*U*CU*AU*CCGCU*GGAAGAU*GGAACCGCU*GGAGAGCAACU*GCAU*AAGGCU*AU*GAAGAGAU*ACGCCCU*GGU*U*CC
U*GGAACAAU*U*GCU*U*U*U*ACAGAU*GCACAU*AU*CGAGGU*GGACAU*CACU*U*ACGCU*GAGU*ACU*U*CGAAAU*GU*CCGU*U*CGGU*U*GGCAGAAGCU*AU*GAAACGAU*AU*GGGC
U*GAAU*ACAAAU*CACAGAAU*CGU*CGU*AU*GCAGU*GAAAACU*CU*CU*U*CAAU*U*CU*U*U*AU*GCCGGU*GU*U*GGGCGCGU*U*AU*U*U*AU*CGGAGU*U*GCAGU*U*GCGCCCGCG
AACGACAU*U*U*AU*AAU*GAACGU*GAAU*U*GCU*CAACAGU*AU*GGGCAU*U*U*CGCAGCCU*ACCGU*GGU*GU*U*CGU*U*U*CCAAAAAGGGGU*U*GCAAAAAAU*U*U*U*GAACGU*GC
AAAAAAAGCU*CCCAAU*CAU*CCAAAAAAU*U*AU*U*AU*CAU*GGAU*U*CU*AAAACGGAU*U*ACCAGGGAU*U*U*CAGU*CGAU*GU*ACACGU*U*CGU*CACAU*CU*CAU*CU*ACCU*CCC
GGU*U*U*U*AAU*GAAU*ACGAU*U*U*U*GU*GCCAGAGU*CCU*U*CGAU*AGGGACAAGACAAU*U*GCACU*GAU*CAU*GAACU*CCU*CU*GGAU*CU*ACU*GGU*CU*GCCU*AAGGGU*GU*
CGCU*CU*GCCU*CAU*AGAACU*GCCU*GCGU*GAGAU*U*CU*CGCAU*GCCAGAGAU*CCU*AU*U*U*U*U*GGCAAU*CAAAU*CAU*U*CCGGAU*ACU*GCGAU*U*U*U*AAGU*GU*U*GU*U
*CCAU*U*CCAU*CACGGU*U*U*U*GGAAU*GU*U*U*ACU*ACACU*CGGAU*AU*U*U*GAU*AU*GU*GGAU*U*U*CGAGU*CGU*CU*U*AAU*GU*AU*AGAU*U*U*GAAGAAGAGCU*GU*U*
U*CU*GAGGAGCCU*U*CAGGAU*U*ACAAGAU*U*CAAAGU*GCGCU*GCU*GGU*GCCAACCCU*AU*U*CU*CCU*U*CU*U*CGCCAAAAGCACU*CU*GAU*U*GACAAAU*ACGAU*U*U*AU*CU
*AAU*U*U*ACACGAAAU*U*GCU*U*CU*GGU*GGCGCU*CCCCU*CU*CU*AAGGAAGU*CGGGGAAGCGGU*U*GCCAAGAGGU*U*CCAU*CU*GCCAGGU*AU*CAGGCAAGGAU*AU*GGGCU*CA
CU*GAGACU*ACAU*CAGCU*AU*U*CU*GAU*U*ACACCCGAGGGGGAU*GAU*AAACCGGGCGCGGU*CGGU*AAAGU*U*GU*U*CCAU*U*U*U*U*U*GAAGCGAAGGU*U*GU*GGAU*CU*GGAU
*ACCGGGAAAACGCU*GGGCGU*U*AAU*CAAAGAGGCGAACU*GU*GU*GU*GAGAGGU*CCU*AU*GAU*U*AU*GU*CCGGU*U*AU*GU*AAACAAU*CCGGAAGCGACCAACGCCU*U*GAU*U*GA
CAAGGAU*GGAU*GGCU*ACAU*U*CU*GGAGACAU*AGCU*U*ACU*GGGACGAAGACGAACACU*U*CU*U*CAU*CGU*U*GACCGCCU*GAAGU*CU*CU*GAU*U*AAGU*ACAAAGGCU*AU*CAG
GU*GGCU*CCCGCU*GAAU*U*GGAAU*CCAU*CU*U*GCU*CCAACACCCCAACAU*CU*U*CGACGCAGGU*GU*CGCAGGU*CU*U*CCCGACGAU*GACGCCGGU*GAACU*U*CCCGCCGCCGU*U*
GU*U*GU*U*U*U*GGAGCACGGAAAGACGAU*GACGGAAAAAGAGAU*CGU*GGAU*U*ACGU*CGCCAGU*CAAGU*AACAACCGCGAAAAAGU*U*GCGCGGAGGAGU*U*GU*GU*U*U*GU*GGACG
AAGU*ACCGAAAGGU*CU*U*ACCGGAAAACU*CGACGCAAGAAAAAU*CAGAGAGAU*CCU*CAU*AAAGGCCAAGAAGGGCGGAAAGAU*CGCCGU*GU*AAU*U*AACAU*AAGCU*AGCU*ACCCAU*
ACGAU*GU*U*CCAGAU*U*ACGCU*CU*CGAGAAAAAAAAAAAAAAAAAAAAAAAAAAAAAAAAAAAAAAAAAAAAAAAAAAAAAAAAAAAAAAAAAAAAAAAAAAAAAAAAAAAAAAAAAAAAAAAAA
AAAAAAAAAAAAAACU*CGAGAU*CG

>Fluc+1FS2

GGAAAU*AAGAGAGAAAAGAAGAGU*AAGAAGAAAU*AU*AACAGCCACCAU*GGGU*GACU*ACAAAGACCAU*GACGGU*GAU*U*AU*AAAGAU*CAU*GACAU*CGAU*U*ACAAGGAU*GACGAU*G
ACAAGCU*CGAAGACGCCAAAAACAU*AAAGAAAGGCCCGGCGCCAU*U*CU*AU*CCGCU*GGAAGAU*GGAACCGCU*GGAGAGCAACU*GCAU*AAGGCU*AU*GAAGAGAU*ACGCCCU*GGU*U*CC
U*GGAACAAU*U*GCU*U*U*U*ACAGAU*GCACAU*AU*CGAGGU*GGACAU*CACU*U*ACGCU*GAGU*ACU*U*CGAAAU*GU*CCGU*U*CGGU*U*GGCAGAAGCU*AU*GAAACGAU*AU*GGGC
U*GAAU*ACAAAU*CACAGAAU*CGU*CGU*AU*GCAGU*GAAAACU*CU*CU*U*CAAU*U*CU*U*U*AU*GCCGGU*GU*U*GGGCGCGU*U*AU*U*U*AU*CGGAGU*U*GCAGU*U*GCGCCCGCG
AACGACAU*U*U*AU*AAU*GAACGU*GAAU*U*GCU*CAACAGU*AU*GGGCAU*U*U*CGCAGCCU*ACCGU*GGU*GU*U*CGU*U*U*CCAAAAAGGGGU*U*GCAAAAAAU*U*U*U*GAACGU*GC
AAAAAAAGCU*CCCAAU*CAU*CCAAAAAAU*U*AU*U*AU*CAU*GGAU*U*CU*AAAACGGAU*U*ACCAGGGAU*U*U*CAGU*CGAU*GU*ACACGU*U*CGU*CACAU*CU*CAU*CU*ACCU*CCC
GGU*U*U*U*AAU*GAAU*ACGAU*U*U*U*GU*GCCAGAGU*CCU*U*CGAU*AGGGACAAGACAAU*U*GCACU*GAU*CAU*GAACU*CCU*CU*GGAU*CU*ACU*GGU*CU*GCCU*AAGGGU*GU*
CGCU*CU*GCCU*CAU*AGAACU*GCCU*GCGU*GAGAU*U*CU*CGCAU*GCCAGAGAU*CCU*AU*U*U*U*U*CGGCAAU*CAAAU*CAU*U*CCGGAU*ACU*GCGAU*U*U*U*AAGU*GU*U*GU*
U*CCAU*U*CCAU*CACGGU*U*U*U*GGAAU*GU*U*U*ACU*ACACU*CGGAU*AU*U*U*GAU*AU*GU*GGAU*U*U*CGAGU*CGU*CU*U*AAU*GU*AU*AGAU*U*U*GAAGAAGAGCU*GU*U
*U*CU*GAGGAGCCU*U*CAGGAU*U*ACAAGAU*U*CAAAGU*GCGCU*GCU*GGU*GCCAACCCU*AU*U*CU*CCU*U*CU*U*CGCCAAAAGCACU*CU*GAU*U*GACAAAU*ACGAU*U*U*AU*C
U*AAU*U*U*ACACGAAAU*U*GCU*U*CU*GGU*GGCGCU*CCCCU*CU*CU*AAGGAAGU*CGGGGAAGCGGU*U*GCCAAGAGGU*U*CCAU*CU*GCCAGGU*AU*CAGGCAAGGAU*AU*GGGCU*C
ACU*GAGACU*ACAU*CAGCU*AU*U*CU*GAU*U*ACACCCGAGGGGGAU*GAU*AAACCGGGCGCGGU*CGGU*AAAGU*U*GU*U*CCAU*U*U*U*U*U*GAAGCGAAGGU*U*GU*GGAU*CU*GGA
U*ACCGGGAAAACGCU*GGGCGU*U*AAU*CAAAGAGGCGAACU*GU*GU*GU*GAGAGGU*CCU*AU*GAU*U*AU*GU*CCGGU*U*AU*GU*AAACAAU*CCGGAAGCGACCAACGCCU*U*GAU*U*G
ACAAGGAU*GGAU*GGCU*ACAU*U*CU*GGAGACAU*AGCU*U*ACU*GGGACGAAGACGAACACU*U*CU*U*CAU*CGU*U*GACCGCCU*GAAGU*CU*CU*GAU*U*AAGU*ACAAAGGCU*AU*CA
GGU*GGCU*CCCGCU*GAAU*U*GGAAU*CCAU*CU*U*GCU*CCAACACCCCAACAU*CU*U*CGACGCAGGU*GU*CGCAGGU*CU*U*CCCGACGAU*GACGCCGGU*GAACU*U*CCCGCCGCCGU*U
*GU*U*GU*U*U*U*GGAGCACGGAAAGACGAU*GACGGAAAAAGAGAU*CGU*GGAU*U*ACGU*CGCCAGU*CAAGU*AACAACCGCGAAAAAGU*U*GCGCGGAGGAGU*U*GU*GU*U*U*GU*GGAC
GAAGU*ACCGAAAGGU*CU*U*ACCGGAAAACU*CGACGCAAGAAAAAU*CAGAGAGAU*CCU*CAU*AAAGGCCAAGAAGGGCGGAAAGAU*CGCCGU*GU*AAU*U*AACAU*AAGCU*AGCU*ACCCAU
*ACGAU*GU*U*CCAGAU*U*ACGCU*CU*CGAGAAAAAAAAAAAAAAAAAAAAAAAAAAAAAAAAAAAAAAAAAAAAAAAAAAAAAAAAAAAAAAAAAAAAAAAAAAAAAAAAAAAAAAAAAAAAAAAA
AAAAAAAAAAAAAAACU*CGAGAU*CG

>A206G
GGAAAU*AAGAGAGAAAAGAAGAGU*AAGAAGAAAU*AU*AACAGCCACCAU*GGGU*GACU*ACAAAGACCAU*GACGGU*GAU*U*AU*AAAGAU*CAU*GACAU*CGAU*U*ACA
AGGAU*GACGAU*GACAAGCU*CGAAGACGCCAAAAACAU*AAAGAAAGGCCCGGCGCCAU*U*CU*AU*CCGCU*GGAAGAU*GGAACCGCU*GGAGAGCAACU*GCAU*AAGGCU*
AU*GAAGAGAU*ACGCCCU*GGU*U*CCU*GGAACAAU*U*GCU*U*U*U*ACAGAU*GCACAU*AU*CGAGGU*GGACAU*CACU*U*ACGCU*GAGU*ACU*U*CGAAAU*GU*CC
GU*U*CGGU*U*GGCAGAAGCU*AU*GAAACGAU*AU*GGGCU*GAAU*ACAAAU*CACAGAAU*CGU*CGU*AU*GCAGU*GAAAACU*CU*CU*U*CAAU*U*CU*U*U*AU*GCC
GGU*GU*U*GGGCGCGU*U*AU*U*U*AU*CGGAGU*U*GCAGU*U*GCGCCCGCGAACGACAU*U*U*AU*AAU*GAACGU*GAAU*U*GCU*CAACAGU*AU*GGGCAU*U*U*CG
CAGCCU*ACCGU*GGU*GU*U*CGU*U*U*CCAAAAAGGGGU*U*GCAAAAAAU*U*U*U*GAACGU*GCAAAAAAAGCU*CCCAAU*CAU*CCAAAAAAU*U*AU*U*AU*CAU*GG
AU*U*CU*AAAACGGAU*U*ACCAGGGAU*U*U*CAGU*CGAU*GU*ACACGU*U*CGU*CACAU*CU*CAU*CU*ACCU*CCCGGU*U*U*U*AAU*GAAU*ACGAU*U*U*U*GU*
GCCAGAGU*CCU*U*CGAU*AGGGACAAGACAAU*U*GCACU*GAU*CAU*GAACU*CCU*CU*GGAU*CU*ACU*GGU*CU*GCCU*AAGGGU*GU*CGCU*CU*GCCU*CAU*AGA
ACU*GCCU*GCGU*GAGAU*U*CU*CGCAU*GCCAGAGAU*CCU*AU*U*U*U*U*CGGCAAU*CAAAU*CAU*U*CCGGAU*ACU*GCGAU*U*U*U*AAGU*GU*U*GU*U*CCAU
*U*CCAU*CACGGU*U*U*U*GGAAU*GU*U*U*ACU*ACACU*CGGAU*AU*U*U*GAU*AU*GU*GGAU*U*U*CGAGU*CGU*CU*U*AAU*GU*AU*AGAU*U*U*GAAGAAGA
GCU*GU*U*U*CU*GAGGAGCCU*U*CAGGAU*U*ACAAGAU*U*CAAAGU*GCGCU*GCU*GGU*GCCAACCCU*AU*U*CU*CCU*U*CU*U*CGCCAAAAGCACU*CU*GAU*U*
GACAAAU*ACGAU*U*U*AU*CU*AAU*U*U*ACACGAAAU*U*GCU*U*CU*GGU*GGCGCU*CCCCU*CU*CU*AAGGAAGU*CGGGGAAGCGGU*U*GCCAAGAGGU*U*CCAU*
CU*GCCAGGU*AU*CAGGCAAGGAU*AU*GGGCU*CACU*GAGACU*ACAU*CAGCU*AU*U*CU*GAU*U*ACACCCGAGGGGGAU*GAU*AAACCGGGCGCGGU*CGGU*AAAGU*
U*GU*U*CCAU*U*U*U*U*U*GAAGCGAAGGU*U*GU*GGAU*CU*GGAU*ACCGGGAAAACGCU*GGGCGU*U*AAU*CAAAGAGGCGAACU*GU*GU*GU*GAGAGGU*CCU*AU
*GAU*U*AU*GU*CCGGU*U*AU*GU*AAACAAU*CCGGAAGCGACCAACGCCU*U*GAU*U*GACAAGGAU*GGAU*GGCU*ACAU*U*CU*GGAGACAU*AGCU*U*ACU*GGGAC
GAAGACGAACACU*U*CU*U*CAU*CGU*U*GACCGCCU*GAAGU*CU*CU*GAU*U*AAGU*ACAAAGGCU*AU*CAGGU*GGCU*CCCGCU*GAAU*U*GGAAU*CCAU*CU*U*G
CU*CCAACACCCCAACAU*CU*U*CGACGCAGGU*GU*CGCAGGU*CU*U*CCCGACGAU*GACGCCGGU*GAACU*U*CCCGCCGCCGU*U*GU*U*GU*U*U*U*GGAGCACGGAA
AGACGAU*GACGGAAAAAGAGAU*CGU*GGAU*U*ACGU*CGCCAGU*CAAGU*AACAACCGCGAAAAAGU*U*GCGCGGAGGAGU*U*GU*GU*U*U*GU*GGACGAAGU*ACCGAA
AGGU*CU*U*ACCGGAAAACU*CGACGCAAGAAAAAU*CAGAGAGAU*CCU*CAU*AAAGGCCAAGAAGGGCGGAAAGAU*CGCCGU*GU*AAU*U*AACAU*AAGCU*AGCU*ACCC
AU*ACGAU*GU*U*CCAGAU*U*ACGCU*CU*CGAGAAAAAAAAAAAAAAAAAAAAAAAAAAAAAAAAAAAAAAAAAAAAAAAAAAAAAAAAAAAAAAAAAAAAAAAAAAAAAAAAAA
AAAAAAAAAAAAAAAAAAAAAAAAAAAAAAACU*CGAGAU*CG

>U*187C
GGAAAU*AAGAGAGAAAAGAAGAGU*AAGAAGAAAU*AU*AACAGCCACCAU*GGGU*GACU*ACAAAGACCAU*GACGGU*GAU*U*AU*AAAGAU*CAU*GACAU*CGAU*U*ACA
AGGAU*GACGAU*GACAAGCU*CGAAGACGCCAAAAACAU*AAAGAAAGGCCCGGCGCCAU*U*CU*AU*CCGCU*GGAAGAU*GGAACCGCU*GGAGAGCAACU*GCAU*AAGGCU*
AU*GAAGAGAU*ACGCCCU*GGU*U*CCU*GGAACAAU*U*GCU*U*U*U*ACAGAU*GCACAU*AU*CGAGGU*GGACAU*CACU*U*ACGCU*GAGU*ACU*U*CGAAAU*GU*CC
GU*U*CGGU*U*GGCAGAAGCU*AU*GAAACGAU*AU*GGGCU*GAAU*ACAAAU*CACAGAAU*CGU*CGU*AU*GCAGU*GAAAACU*CU*CU*U*CAAU*U*CU*U*U*AU*GCC
GGU*GU*U*GGGCGCGU*U*AU*U*U*AU*CGGAGU*U*GCAGU*U*GCGCCCGCGAACGACAU*U*U*AU*AAU*GAACGU*GAAU*U*GCU*CAACAGU*AU*GGGCAU*U*U*CG
CAGCCU*ACCGU*GGU*GU*U*CGU*U*U*CCAAAAAGGGGU*U*GCAAAAAAU*U*U*U*GAACGU*GCAAAAAAAGCU*CCCAAU*CAU*CCAAAAAAU*U*AU*U*AU*CAU*GG
AU*U*CU*AAAACGGAU*U*ACCAGGGAU*U*U*CAGU*CGAU*GU*ACACGU*U*CGU*CACAU*CU*CAU*CU*ACCU*CCCGGU*U*U*U*AAU*GAAU*ACGAU*U*U*U*GU*
GCCAGAGU*CCU*U*CGAU*AGGGACAAGACAAU*U*GCACU*GAU*CAU*GAACU*CCU*CU*GGAU*CU*ACU*GGU*CU*GCCU*AAAGGU*GU*CGCU*CU*GCCU*CAU*AGA
ACU*GCCU*GCGU*GAGAU*U*CU*CGCAU*GCCAGAGAU*CCU*AU*U*U*U*CCGGCAAU*CAAAU*CAU*U*CCGGAU*ACU*GCGAU*U*U*U*AAGU*GU*U*GU*U*CCAU*
U*CCAU*CACGGU*U*U*U*GGAAU*GU*U*U*ACU*ACACU*CGGAU*AU*U*U*GAU*AU*GU*GGAU*U*U*CGAGU*CGU*CU*U*AAU*GU*AU*AGAU*U*U*GAAGAAGAG
CU*GU*U*U*CU*GAGGAGCCU*U*CAGGAU*U*ACAAGAU*U*CAAAGU*GCGCU*GCU*GGU*GCCAACCCU*AU*U*CU*CCU*U*CU*U*CGCCAAAAGCACU*CU*GAU*U*G
ACAAAU*ACGAU*U*U*AU*CU*AAU*U*U*ACACGAAAU*U*GCU*U*CU*GGU*GGCGCU*CCCCU*CU*CU*AAGGAAGU*CGGGGAAGCGGU*U*GCCAAGAGGU*U*CCAU*C
U*GCCAGGU*AU*CAGGCAAGGAU*AU*GGGCU*CACU*GAGACU*ACAU*CAGCU*AU*U*CU*GAU*U*ACACCCGAGGGGGAU*GAU*AAACCGGGCGCGGU*CGGU*AAAGU*U
*GU*U*CCAU*U*U*U*U*U*GAAGCGAAGGU*U*GU*GGAU*CU*GGAU*ACCGGGAAAACGCU*GGGCGU*U*AAU*CAAAGAGGCGAACU*GU*GU*GU*GAGAGGU*CCU*AU*
GAU*U*AU*GU*CCGGU*U*AU*GU*AAACAAU*CCGGAAGCGACCAACGCCU*U*GAU*U*GACAAGGAU*GGAU*GGCU*ACAU*U*CU*GGAGACAU*AGCU*U*ACU*GGGACG
AAGACGAACACU*U*CU*U*CAU*CGU*U*GACCGCCU*GAAGU*CU*CU*GAU*U*AAGU*ACAAAGGCU*AU*CAGGU*GGCU*CCCGCU*GAAU*U*GGAAU*CCAU*CU*U*GC
U*CCAACACCCCAACAU*CU*U*CGACGCAGGU*GU*CGCAGGU*CU*U*CCCGACGAU*GACGCCGGU*GAACU*U*CCCGCCGCCGU*U*GU*U*GU*U*U*U*GGAGCACGGAAA
GACGAU*GACGGAAAAAGAGAU*CGU*GGAU*U*ACGU*CGCCAGU*CAAGU*AACAACCGCGAAAAAGU*U*GCGCGGAGGAGU*U*GU*GU*U*U*GU*GGACGAAGU*ACCGAAA
GGU*CU*U*ACCGGAAAACU*CGACGCAAGAAAAAU*CAGAGAGAU*CCU*CAU*AAAGGCCAAGAAGGGCGGAAAGAU*CGCCGU*GU*AAU*U*AACAU*AAGCU*AGCU*ACCCA
U*ACGAU*GU*U*CCAGAU*U*ACGCU*CU*CGAGAAAAAAAAAAAAAAAAAAAAAAAAAAAAAAAAAAAAAAAAAAAAAAAAAAAAAAAAAAAAAAAAAAAAAAAAAAAAAAAAAA
AAAAAAAAAAAAAAAAAAAAAAAAAAAAAACU*CGAGAU*CG

>U*208C
GGAAAU*AAGAGAGAAAAGAAGAGU*AAGAAGAAAU*AU*AACAGCCACCAU*GGGU*GACU*ACAAAGACCAU*GACGGU*GAU*U*AU*AAAGAU*CAU*GACAU*CGAU*U*ACA
AGGAU*GACGAU*GACAAGCU*CGAAGACGCCAAAAACAU*AAAGAAAGGCCCGGCGCCAU*U*CU*AU*CCGCU*GGAAGAU*GGAACCGCU*GGAGAGCAACU*GCAU*AAGGCU*
AU*GAAGAGAU*ACGCCCU*GGU*U*CCU*GGAACAAU*U*GCU*U*U*U*ACAGAU*GCACAU*AU*CGAGGU*GGACAU*CACU*U*ACGCU*GAGU*ACU*U*CGAAAU*GU*CC
GU*U*CGGU*U*GGCAGAAGCU*AU*GAAACGAU*AU*GGGCU*GAAU*ACAAAU*CACAGAAU*CGU*CGU*AU*GCAGU*GAAAACU*CU*CU*U*CAAU*U*CU*U*U*AU*GCC
GGU*GU*U*GGGCGCGU*U*AU*U*U*AU*CGGAGU*U*GCAGU*U*GCGCCCGCGAACGACAU*U*U*AU*AAU*GAACGU*GAAU*U*GCU*CAACAGU*AU*GGGCAU*U*U*CG
CAGCCU*ACCGU*GGU*GU*U*CGU*U*U*CCAAAAAGGGGU*U*GCAAAAAAU*U*U*U*GAACGU*GCAAAAAAAGCU*CCCAAU*CAU*CCAAAAAAU*U*AU*U*AU*CAU*GG
AU*U*CU*AAAACGGAU*U*ACCAGGGAU*U*U*CAGU*CGAU*GU*ACACGU*U*CGU*CACAU*CU*CAU*CU*ACCU*CCCGGU*U*U*U*AAU*GAAU*ACGAU*U*U*U*GU*
GCCAGAGU*CCU*U*CGAU*AGGGACAAGACAAU*U*GCACU*GAU*CAU*GAACU*CCU*CU*GGAU*CU*ACU*GGU*CU*GCCU*AAAGGU*GU*CGCU*CU*GCCU*CAU*AGA
ACU*GCCU*GCGU*GAGAU*U*CU*CGCAU*GCCAGAGAU*CCU*AU*U*U*U*U*CGGCAAU*CAAAU*CAU*U*CCGGAU*ACU*GCGAU*U*U*U*AAGU*GU*U*GU*U*CCAU
*U*CCAU*CACGGU*U*CU*GGAAU*GU*U*U*ACU*ACACU*CGGAU*AU*U*U*GAU*AU*GU*GGAU*U*U*CGAGU*CGU*CU*U*AAU*GU*AU*AGAU*U*U*GAAGAAGAG
CU*GU*U*U*CU*GAGGAGCCU*U*CAGGAU*U*ACAAGAU*U*CAAAGU*GCGCU*GCU*GGU*GCCAACCCU*AU*U*CU*CCU*U*CU*U*CGCCAAAAGCACU*CU*GAU*U*G
ACAAAU*ACGAU*U*U*AU*CU*AAU*U*U*ACACGAAAU*U*GCU*U*CU*GGU*GGCGCU*CCCCU*CU*CU*AAGGAAGU*CGGGGAAGCGGU*U*GCCAAGAGGU*U*CCAU*C
U*GCCAGGU*AU*CAGGCAAGGAU*AU*GGGCU*CACU*GAGACU*ACAU*CAGCU*AU*U*CU*GAU*U*ACACCCGAGGGGGAU*GAU*AAACCGGGCGCGGU*CGGU*AAAGU*U
*GU*U*CCAU*U*U*U*U*U*GAAGCGAAGGU*U*GU*GGAU*CU*GGAU*ACCGGGAAAACGCU*GGGCGU*U*AAU*CAAAGAGGCGAACU*GU*GU*GU*GAGAGGU*CCU*AU*
GAU*U*AU*GU*CCGGU*U*AU*GU*AAACAAU*CCGGAAGCGACCAACGCCU*U*GAU*U*GACAAGGAU*GGAU*GGCU*ACAU*U*CU*GGAGACAU*AGCU*U*ACU*GGGACG
AAGACGAACACU*U*CU*U*CAU*CGU*U*GACCGCCU*GAAGU*CU*CU*GAU*U*AAGU*ACAAAGGCU*AU*CAGGU*GGCU*CCCGCU*GAAU*U*GGAAU*CCAU*CU*U*GC
U*CCAACACCCCAACAU*CU*U*CGACGCAGGU*GU*CGCAGGU*CU*U*CCCGACGAU*GACGCCGGU*GAACU*U*CCCGCCGCCGU*U*GU*U*GU*U*U*U*GGAGCACGGAAA
GACGAU*GACGGAAAAAGAGAU*CGU*GGAU*U*ACGU*CGCCAGU*CAAGU*AACAACCGCGAAAAAGU*U*GCGCGGAGGAGU*U*GU*GU*U*U*GU*GGACGAAGU*ACCGAAA
GGU*CU*U*ACCGGAAAACU*CGACGCAAGAAAAAU*CAGAGAGAU*CCU*CAU*AAAGGCCAAGAAGGGCGGAAAGAU*CGCCGU*GU*AAU*U*AACAU*AAGCU*AGCU*ACCCA
U*ACGAU*GU*U*CCAGAU*U*ACGCU*CU*CGAGAAAAAAAAAAAAAAAAAAAAAAAAAAAAAAAAAAAAAAAAAAAAAAAAAAAAAAAAAAAAAAAAAAAAAAAAAAAAAAAAAA
AAAAAAAAAAAAAAAAAAAAAAAAAAAAAACU*CGAGAU*CG

>U*187C.U*208C
GGAAAU*AAGAGAGAAAAGAAGAGU*AAGAAGAAAU*AU*AACAGCCACCAU*GGGU*GACU*ACAAAGACCAU*GACGGU*GAU*U*AU*AAAGAU*CAU*GACAU*CGAU*U*ACA
AGGAU*GACGAU*GACAAGCU*CGAAGACGCCAAAAACAU*AAAGAAAGGCCCGGCGCCAU*U*CU*AU*CCGCU*GGAAGAU*GGAACCGCU*GGAGAGCAACU*GCAU*AAGGCU*
AU*GAAGAGAU*ACGCCCU*GGU*U*CCU*GGAACAAU*U*GCU*U*U*U*ACAGAU*GCACAU*AU*CGAGGU*GGACAU*CACU*U*ACGCU*GAGU*ACU*U*CGAAAU*GU*CC
GU*U*CGGU*U*GGCAGAAGCU*AU*GAAACGAU*AU*GGGCU*GAAU*ACAAAU*CACAGAAU*CGU*CGU*AU*GCAGU*GAAAACU*CU*CU*U*CAAU*U*CU*U*U*AU*GCC
GGU*GU*U*GGGCGCGU*U*AU*U*U*AU*CGGAGU*U*GCAGU*U*GCGCCCGCGAACGACAU*U*U*AU*AAU*GAACGU*GAAU*U*GCU*CAACAGU*AU*GGGCAU*U*U*CG
CAGCCU*ACCGU*GGU*GU*U*CGU*U*U*CCAAAAAGGGGU*U*GCAAAAAAU*U*U*U*GAACGU*GCAAAAAAAGCU*CCCAAU*CAU*CCAAAAAAU*U*AU*U*AU*CAU*GG
AU*U*CU*AAAACGGAU*U*ACCAGGGAU*U*U*CAGU*CGAU*GU*ACACGU*U*CGU*CACAU*CU*CAU*CU*ACCU*CCCGGU*U*U*U*AAU*GAAU*ACGAU*U*U*U*GU*
GCCAGAGU*CCU*U*CGAU*AGGGACAAGACAAU*U*GCACU*GAU*CAU*GAACU*CCU*CU*GGAU*CU*ACU*GGU*CU*GCCU*AAAGGU*GU*CGCU*CU*GCCU*CAU*AGA
ACU*GCCU*GCGU*GAGAU*U*CU*CGCAU*GCCAGAGAU*CCU*AU*U*U*U*CCGGCAAU*CAAAU*CAU*U*CCGGAU*ACU*GCGAU*U*U*U*AAGU*GU*U*GU*U*CCAU*
U*CCAU*CACGGU*U*CU*GGAAU*GU*U*U*ACU*ACACU*CGGAU*AU*U*U*GAU*AU*GU*GGAU*U*U*CGAGU*CGU*CU*U*AAU*GU*AU*AGAU*U*U*GAAGAAGAGC
U*GU*U*U*CU*GAGGAGCCU*U*CAGGAU*U*ACAAGAU*U*CAAAGU*GCGCU*GCU*GGU*GCCAACCCU*AU*U*CU*CCU*U*CU*U*CGCCAAAAGCACU*CU*GAU*U*GA
CAAAU*ACGAU*U*U*AU*CU*AAU*U*U*ACACGAAAU*U*GCU*U*CU*GGU*GGCGCU*CCCCU*CU*CU*AAGGAAGU*CGGGGAAGCGGU*U*GCCAAGAGGU*U*CCAU*CU
*GCCAGGU*AU*CAGGCAAGGAU*AU*GGGCU*CACU*GAGACU*ACAU*CAGCU*AU*U*CU*GAU*U*ACACCCGAGGGGGAU*GAU*AAACCGGGCGCGGU*CGGU*AAAGU*U*
GU*U*CCAU*U*U*U*U*U*GAAGCGAAGGU*U*GU*GGAU*CU*GGAU*ACCGGGAAAACGCU*GGGCGU*U*AAU*CAAAGAGGCGAACU*GU*GU*GU*GAGAGGU*CCU*AU*G
AU*U*AU*GU*CCGGU*U*AU*GU*AAACAAU*CCGGAAGCGACCAACGCCU*U*GAU*U*GACAAGGAU*GGAU*GGCU*ACAU*U*CU*GGAGACAU*AGCU*U*ACU*GGGACGA
AGACGAACACU*U*CU*U*CAU*CGU*U*GACCGCCU*GAAGU*CU*CU*GAU*U*AAGU*ACAAAGGCU*AU*CAGGU*GGCU*CCCGCU*GAAU*U*GGAAU*CCAU*CU*U*GCU
*CCAACACCCCAACAU*CU*U*CGACGCAGGU*GU*CGCAGGU*CU*U*CCCGACGAU*GACGCCGGU*GAACU*U*CCCGCCGCCGU*U*GU*U*GU*U*U*U*GGAGCACGGAAAG
ACGAU*GACGGAAAAAGAGAU*CGU*GGAU*U*ACGU*CGCCAGU*CAAGU*AACAACCGCGAAAAAGU*U*GCGCGGAGGAGU*U*GU*GU*U*U*GU*GGACGAAGU*ACCGAAAG
GU*CU*U*ACCGGAAAACU*CGACGCAAGAAAAAU*CAGAGAGAU*CCU*CAU*AAAGGCCAAGAAGGGCGGAAAGAU*CGCCGU*GU*AAU*U*AACAU*AAGCU*AGCU*ACCCAU
*ACGAU*GU*U*CCAGAU*U*ACGCU*CU*CGAGAAAAAAAAAAAAAAAAAAAAAAAAAAAAAAAAAAAAAAAAAAAAAAAAAAAAAAAAAAAAAAAAAAAAAAAAAAAAAAAAAAAAAAAA
AAAAAAAAAAAAAAAAAAAAAAAAAAAAACU*CGAGAU*CG

Supplementary Fig. 1: Reporter RNA sequences

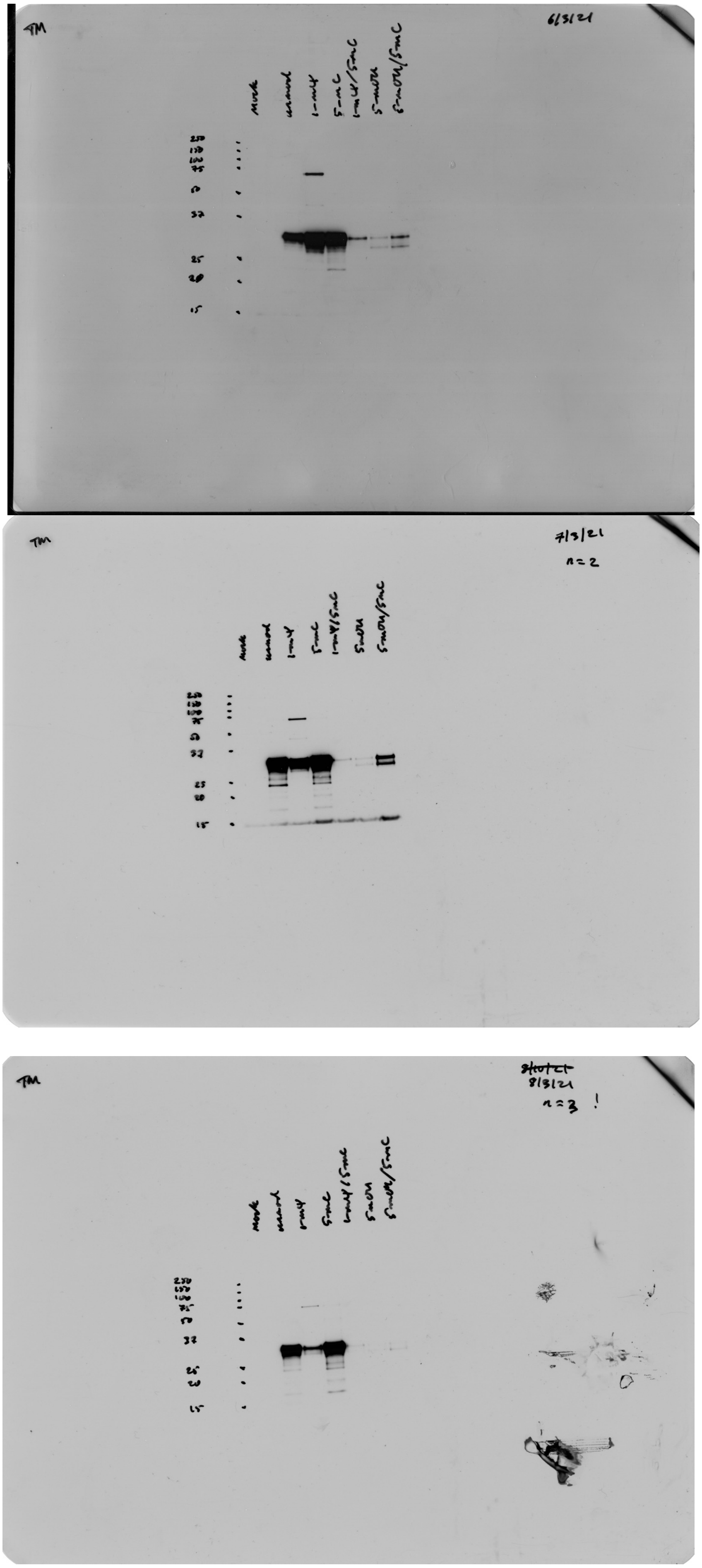

Supplementary Fig. 2: Uncropped films for western blot displayed in Figure 1e

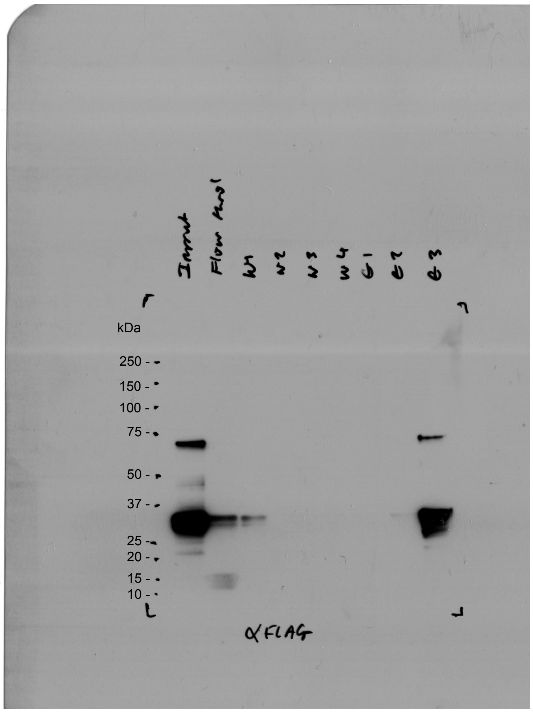

Supplementary Fig 3. Uncropped film for western blot displayed in Fig 3a

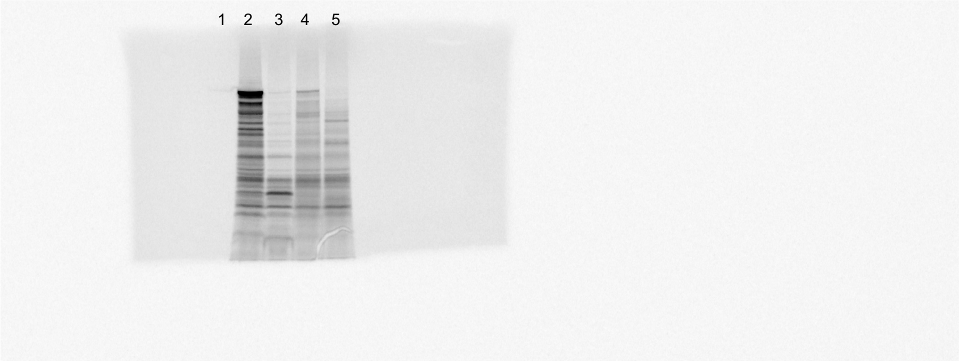

(all)
1. Mock
2. WTFLUC unmodified
3. WTFLUC 1-methylΨ
4. WTFLUC unmodified + paromomycin
5. WTFLUC 1-methylΨ + paromomycin

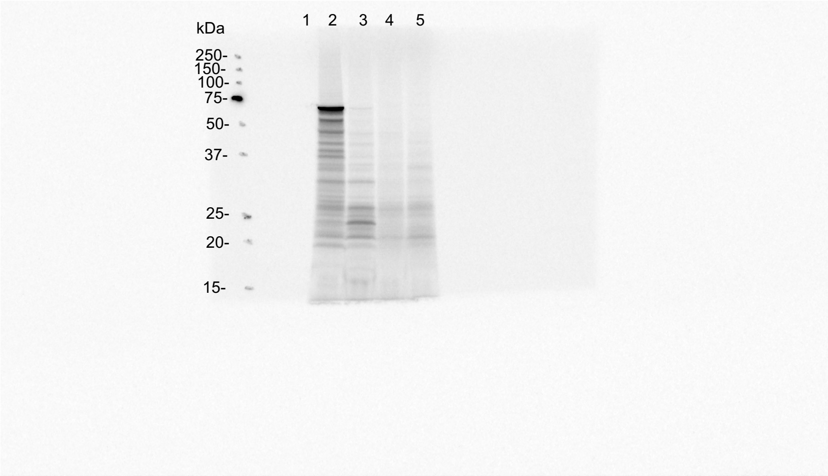

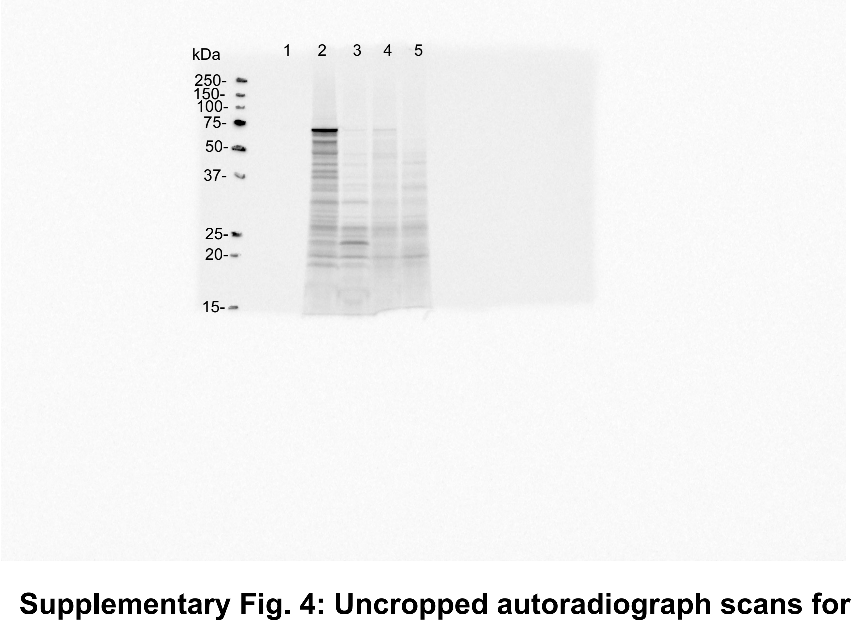

Supplementary Fig. 4: Uncropped autoradiograph scans for autoradiograph displayed in Figure 4a

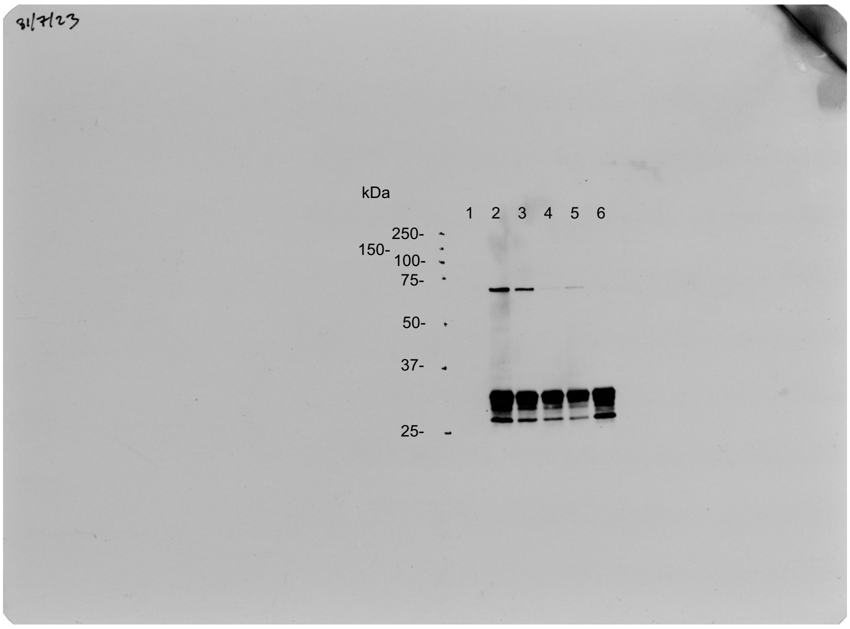

1. Mock
2. Fluc+1FS2
3. A206G
4. T187C
5. T208C
6. T187C.T208C

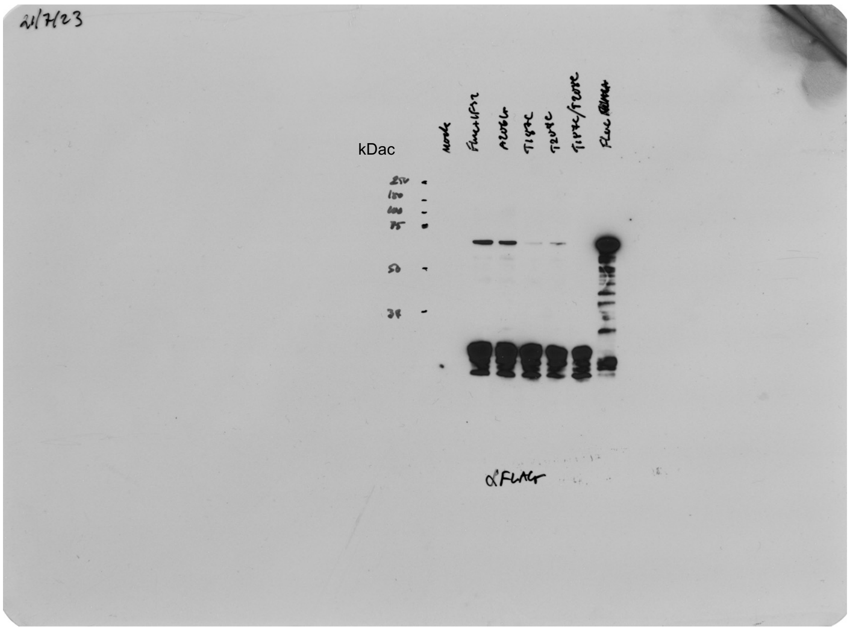

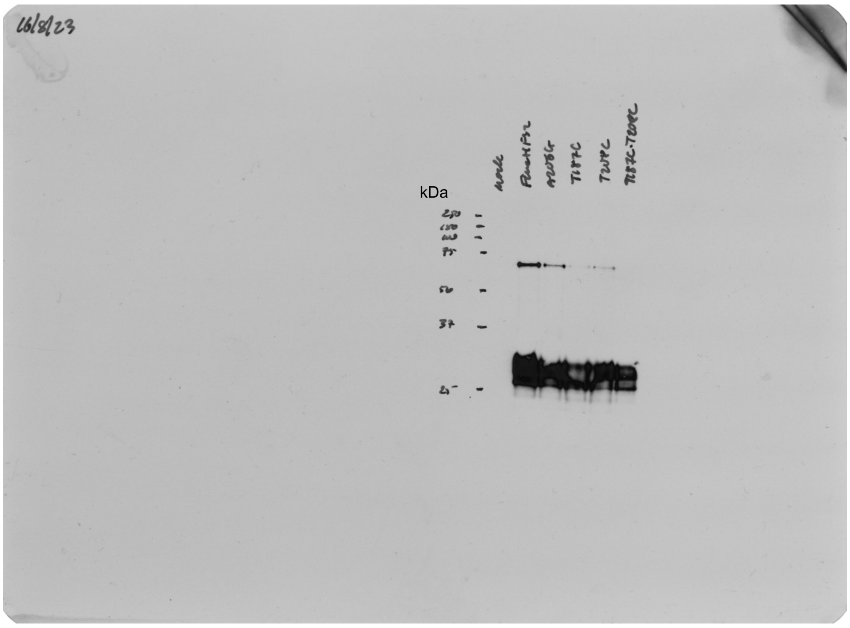

Supplementary Fig. 5: Uncropped films for western blot displayed in Figure 4e

a

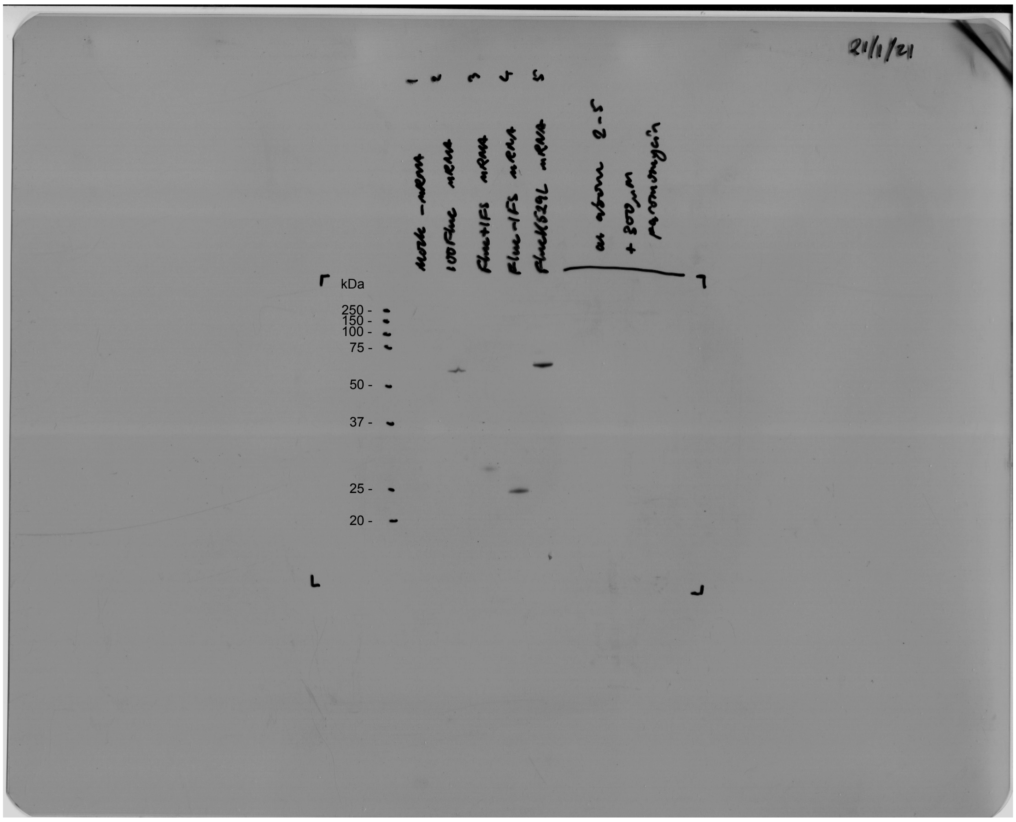

b

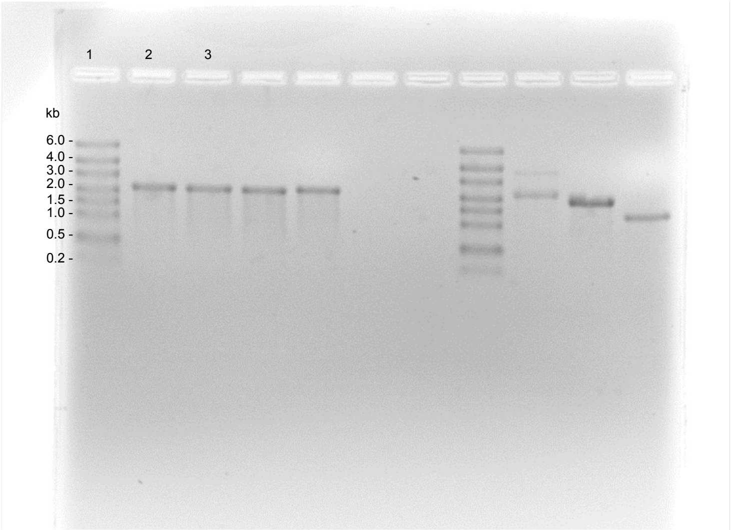

1. RiboRuler HR
2. WTFluc
3. Fluc+1FS

Supplementary Fig. 6. Uncropped images for Extended Data Fig. 1
a, Uncropped film for western blot displayed in Extended Data Fig. 1. **b**, Uncropped UV gel photograph for gel displayed in Extended Fig. 1.

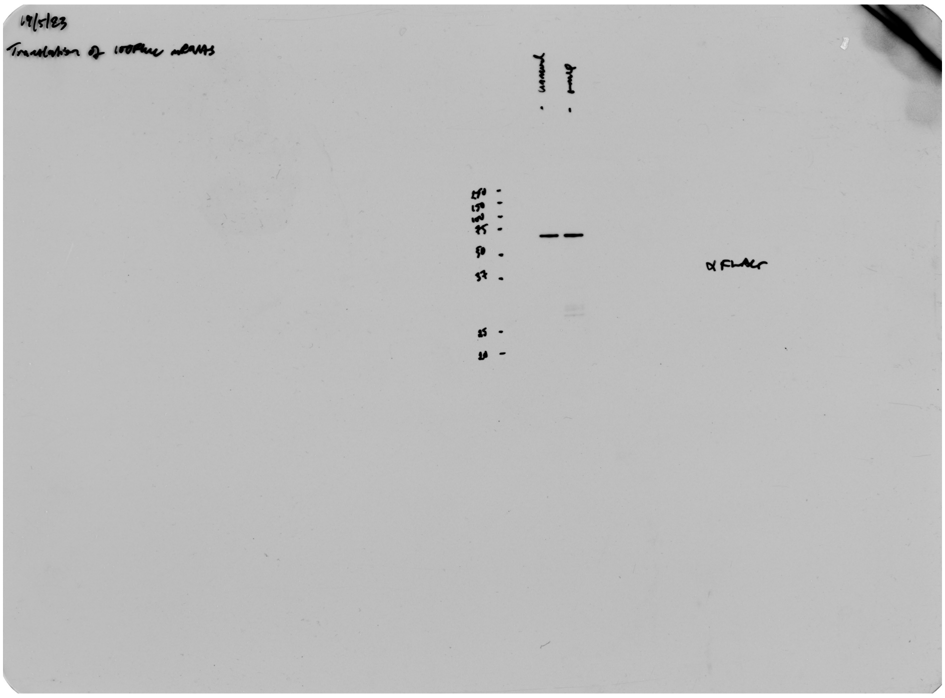

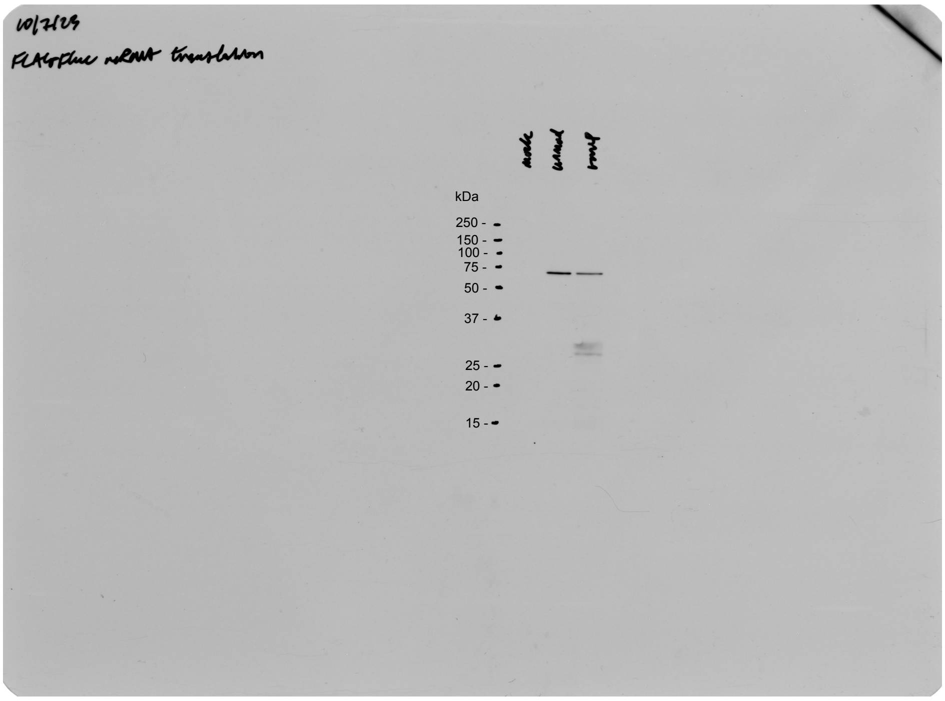

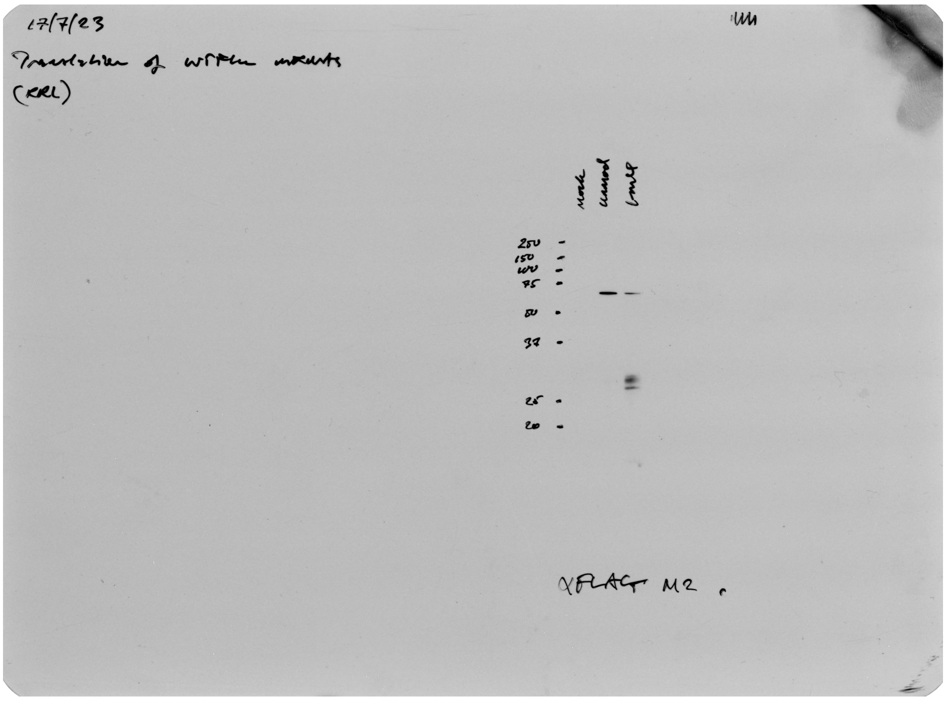

Supplementary Fig. 7: Uncropped films for western blot displayed in Extended Data Figure 5
