# Supplementary material for: N1-methylpseudouridylation of mRNA causes +1 ribosomal frameshifting
Source: Nature. 2023 Dec 6;625(7993):189–94. doi: 10.1038/s41586-023-06800-3 (PMC10764286; doi:10.1038/s41586-023-06800-3)
Supplement: Supplementary file 2 — Reporting Summary [file 41586_2023_6800_MOESM2_ESM.pdf]

nature portfolio

Corresponding author(s): Anne Willis, James Thaventhiran

Last updated by author(s): 13/10/23

Reporting Summary

Nature Portfolio wishes to improve the reproducibility of the work that we publish. This form provides structure for consistency and transparency in reporting. For further information on Nature Portfolio policies, see our Editorial Policies and the Editorial Policy Checklist.

Statistics

For all statistical analyses, confirm that the following items are present in the figure legend, table legend, main text, or Methods section.

n/a	Confirmed	
☐	☒	The exact sample size (*n*) for each experimental group/condition, given as a discrete number and unit of measurement
☐	☒	A statement on whether measurements were taken from distinct samples or whether the same sample was measured repeatedly
☐	☒	The statistical test(s) used AND whether they are one- or two-sided<br>*Only common tests should be described solely by name; describe more complex techniques in the Methods section.*
☒	☐	A description of all covariates tested
☐	☒	A description of any assumptions or corrections, such as tests of normality and adjustment for multiple comparisons
☐	☒	A full description of the statistical parameters including central tendency (e.g. means) or other basic estimates (e.g. regression coefficient) AND variation (e.g. standard deviation) or associated estimates of uncertainty (e.g. confidence intervals)
☐	☒	For null hypothesis testing, the test statistic (e.g. *F*, *t*, *r*) with confidence intervals, effect sizes, degrees of freedom and *P* value noted<br>*Give P values as exact values whenever suitable.*
☒	☐	For Bayesian analysis, information on the choice of priors and Markov chain Monte Carlo settings
☒	☐	For hierarchical and complex designs, identification of the appropriate level for tests and full reporting of outcomes
☒	☐	Estimates of effect sizes (e.g. Cohen's *d*, Pearson's *r*), indicating how they were calculated

Our web collection on statistics for biologists contains articles on many of the points above.

Software and code

Policy information about availability of computer code

Data collection	AID EliSpot Software (version 7.0), Proteome Discoverer (version 2.5).
Data analysis	R (version 4.3.0), ggplot2 (version 3.4.2), DescTools (version 0.99.46), STAR (version 2.7.4a). Scripts for analysis of RNA-seq data are available at: https://github.com/tom-mulroney/rna-seq_mutations.

For manuscripts utilizing custom algorithms or software that are central to the research but not yet described in published literature, software must be made available to editors and reviewers. We strongly encourage code deposition in a community repository (e.g. GitHub). See the Nature Portfolio guidelines for submitting code & software for further information.

Data

Policy information about availability of data

All manuscripts must include a data availability statement. This statement should provide the following information, where applicable:

- Accession codes, unique identifiers, or web links for publicly available datasets
- A description of any restrictions on data availability
- For clinical datasets or third party data, please ensure that the statement adheres to our policy

Mass spectrometry data have been deposited with MassIVE ID MSV000093074. RNA-seq reads and processed files are available at NCBI Gene Expression Omnibus (Accession GSE223044). Additional data are available from Figshare (DOI: 10.6084/m9.figshare.24271744). The following accessions were used for mass spectrometry analysis: UP000001811 and P08659 (UniProt).

Human research participants

Policy information about studies involving human research participants and Sex and Gender in Research.

Reporting on sex and gender	Sex and gender were not considered during study design. 37% of study participants were male. 63% were female. These data are included in Supplementary Table 1.
Population characteristics	Age, sex.
Recruitment	Participants were initially recruited as part of observational studies of the COVID-19 vaccine responses (https://doi.org/10.1016/j.cell.2021.10.011, https://doi.org/10.1038/s41591-023-02343-2, https://doi.org/10.1038/s41467-023-38810-0). Excess available samples from healthy vaccinated participants were subject to analysis. There are no recruitment biases identified that are likely to impact results.
Ethics oversight	Human sample collection and analysis was conducted in accordance with the principles of Good Clinical Practice and following approved protocols of the NIHR National Bioresource. Samples were collected with the written informed consent of all study participants under the NIHR National BioResource-Research Tissue Bank (NBR-RTB) ethics (REC:17/EE/0025) and from the PITCH study. PITCH is a sub-study of the SIREN study, which was approved by the Berkshire Research Ethics Committee, Health Research 250 Authority (IRAS ID 284460, REC reference 20/SC/0230), with PITCH recognised as a sub-study on 2nd December 2020. SIREN is registered with ISRCTN (Trial ID:252 ISRCTN11041050). Some participants were recruited under aligned study protocols. In Liverpool, some participants were recruited under the "Human immune responses to acute virus infections" Study (16/NW/0170), approved by North West - Liverpool Central Research Ethics Committee on 8th March 2016, and amended on 14th September 2020 and 4th May 2021. In Oxford, participants were recruited under the GI Biobank Study 16/YH/0247, approved by the research ethics committee (REC) at Yorkshire & The Humber - Sheffield Research Ethics Committee on 29th July 2016, which has been amended for this purpose on 8th June 2020. The study was conducted in compliance with all relevant ethical regulations for work with human participants, and according to the principles of the Declaration of Helsinki (2008) and the International Conference on Harmonization (ICH) Good Clinical Practice (GCP) guidelines. Written informed consent to publish clinical and genetic data, in addition to study participation was obtained for all participants enrolled in the study.

Note that full information on the approval of the study protocol must also be provided in the manuscript.

Field-specific reporting

Please select the one below that is the best fit for your research. If you are not sure, read the appropriate sections before making your selection.

☒ Life sciences ☐ Behavioural & social sciences ☐ Ecological, evolutionary & environmental sciences

For a reference copy of the document with all sections, see nature.com/documents/nr-reporting-summary-flat.pdf

Life sciences study design

All studies must disclose on these points even when the disclosure is negative.

Sample size	Sample sizes for mouse experiments were determined based on previous analysis. Because antigen-specific CD8+ T cell numbers are likely to be a continuous trait, the widely-accepted formula for calculating sample sizes for continuous variable published by Snedecor and Cochran (1989) has been used (https://doi.org/10.1017/S0021859600074104), with the following variable (s = standard deviation (~10% of mean - per previous mouse experiments in the lab), d = desired effect size to detect (± 15% of mean), C = constant dependent on the value of α and β (in this case 7.85; see https://doi.org/10.1093/ilar.43.4.207, α = 0.05, β = 0.2 (so that power = 80% i.e. 1 - β)). So: n = 1 + ((2 x 7.85) x (0.1/0.15)2) = 7.97 (i.e. 8 in each sample group). Human sample sizes were limited due to available excess PBMC samples from recruited study participants of studies of the COVID-19 vaccine response (https://doi.org/10.1016/j.cell.2021.10.011, https://doi.org/10.1038/s41591-023-02343-2, https://doi.org/10.1038/s41467-023-38810-0). A power analysis calculation for available samples was performed using MESS R package (version 0.5.9) prior to analysis based on effect sizes from previous experiments for n=20, n=21 (2-groups, one-tail, unequal variance, p=0.05), yielding power>0.8. Sample sizes for in vitro experiments were determined by previous similar experiments (e.g. https://doi.org/10.1093/nar/gkq347).
Data exclusions	No data were excluded from analysis.
Replication	All attempts at replication are included in the figures.
Randomization	Randomisation was not applicable at this was a non-interventional study.
Blinding	Blinding was not applicable at this was a non-interventional study.

Reporting for specific materials, systems and methods

We require information from authors about some types of materials, experimental systems and methods used in many studies. Here, indicate whether each material, system or method listed is relevant to your study. If you are not sure if a list item applies to your research, read the appropriate section before selecting a response.

Materials & experimental systems

n/a	Involved in the study
☐	☒ Antibodies
☐	☒ Eukaryotic cell lines
☒	☐ Palaeontology and archaeology
☐	☒ Animals and other organisms
☒	☐ Clinical data
☒	☐ Dual use research of concern

Methods

n/a	Involved in the study
☒	☐ ChIP-seq
☒	☐ Flow cytometry
☒	☐ MRI-based neuroimaging

Antibodies

Antibodies used	anti-FLAG M2 (Primary antibody raised in mouse)(Sigma Aldrich F1804), anti-mouse-HRP (secondary antibody raised in goat)(Dako P0447), anti-FLAG magnetic agarose (primary antibody conjugate raised in rat, Thermo Scientific A36797), IFNγ detector antibody clone 7-B6-1 (included in MabTech 3420-4APT kit).
Validation	anti-FLAG M2 antibody (Sigma Aldrich F1804) has been extensively validated by previous studies and the manufacturer for western blotting (e.g. https://doi.org/10.1186/s12985-016-0610-7, https://doi.org/10.1016/j.bbamcr.2019.06.002.). anti-FLAG magnetic agarose (Thermo Scientific A36797) has been validated for immunoprecipitation (e.g. https://doi.org/10.1002/cpz1.156). IFNγ detector antibody clone 7-B6-1 has been validated for ELISpot (e.g. https://doi.org/10.1016/j.cell.2020.08.017.).

Eukaryotic cell lines

Policy information about cell lines and Sex and Gender in Research

Cell line source(s)	HeLa cells (sex female) were obtained from ATCC.
Authentication	HeLa cells were independently authenticated by STR typing.
Mycoplasma contamination	HeLa cells were tested for mycoplasma infection and tested negative for mycoplasma infection.
Commonly misidentified lines (See ICLAC register)	HeLa cells are not a Misidentified Cell Line according to ICLAC register Version 12 (2023).

Animals and other research organisms

Policy information about studies involving animals; ARRIVE guidelines recommended for reporting animal research, and Sex and Gender in Research

Laboratory animals	C57BL/6J mice (wild type, WT) were purchased from Charles River laboratories. Mice were used at 8-12 weeks age.<br>Mice were housed at the University of Cambridge as specific pathogen-free/SPF, 19-23 degrees Celsius, and the humidity is kept 45%-65%, a 12 hour (7am- 7pm) light dark cycle.
Wild animals	No wild animals were used in the study.
Reporting on sex	Sex was not considered in the study design. All mice were female.
Field-collected samples	No field collected samples were used in the study.
Ethics oversight	Animal experiments were licensed by the UK Home Office according to the Animals Scientific Procedures Act 1986 (License PP6047951), approved and conducted in compliance with protocols by the University of Cambridge, University Biomedical Services Animal Welfare and Ethical Review Bodies (AWERB) committee.

Note that full information on the approval of the study protocol must also be provided in the manuscript.
